# The Di-Iron Protein YtfE Is a Nitric Oxide-Generating Nitrite Reductase Involved in the Management of Nitrosative Stress

**DOI:** 10.1021/jacs.1c12407

**Published:** 2022-04-13

**Authors:** Jason C. Crack, Basema K. Balasiny, Sophie P. Bennett, Matthew D. Rolfe, Afonso Froes, Fraser MacMillan, Jeffrey Green, Jeffrey A. Cole, Nick E. Le Brun

**Affiliations:** † Centre for Molecular and Structural Biochemistry, School of Chemistry, University of East Anglia, Norwich Research Park, Norwich NR4 7TJ, UK; ‡ Institute of Microbiology and Infection and School of Biosciences, University of Birmingham, Birmingham B15 2TT, UK; § School of Biosciences, University of Sheffield, Sheffield S10 2TN, UK

## Abstract

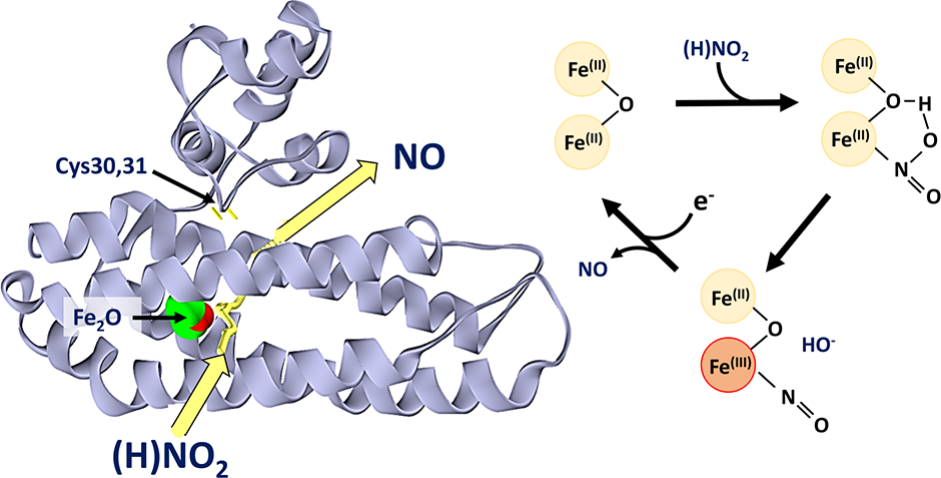

Previously characterized nitrite reductases fall into three classes: siroheme-containing enzymes (NirBD), cytochrome *c* hemoproteins (NrfA and NirS), and copper-containing enzymes (NirK). We show here that the di-iron protein YtfE represents a physiologically relevant new class of nitrite reductases. Several functions have been previously proposed for YtfE, including donating iron for the repair of iron–sulfur clusters that have been damaged by nitrosative stress, releasing nitric oxide (NO) from nitrosylated iron, and reducing NO to nitrous oxide (N_2_O). Here, *in vivo* reporter assays confirmed that *Escherichia coli* YtfE increased cytoplasmic NO production from nitrite. Spectroscopic and mass spectrometric investigations revealed that the di-iron site of YtfE exists in a mixture of forms, including nitrosylated and nitrite-bound, when isolated from nitrite-supplemented, but not nitrate-supplemented, cultures. Addition of nitrite to di-ferrous YtfE resulted in nitrosylated YtfE and the release of NO. Kinetics of nitrite reduction were dependent on the nature of the reductant; the lowest *K*_m_, measured for the di-ferrous form, was ∼90 μM, well within the intracellular nitrite concentration range. The vicinal di-cysteine motif, located in the N-terminal domain of YtfE, was shown to function in the delivery of electrons to the di-iron center. Notably, YtfE exhibited very low NO reductase activity and was only able to act as an iron donor for reconstitution of apo-ferredoxin under conditions that damaged its di-iron center. Thus, YtfE is a high-affinity, low-capacity nitrite reductase that we propose functions to relieve nitrosative stress by acting in combination with the co-regulated NO-consuming enzymes Hmp and Hcp.

## Introduction

The Enterobacteriaceae genera of γ-proteobacteria are a diverse family of commensal, pathogenic, and saprophytic species found in animal intestinal tracts and aquatic or terrestrial environments. They are facultative anaerobes, many of which can, in the absence of O_2_, preferentially use environmental nitrate (NO_3_^–^) or nitrite (NO_2_^–^) as a terminal respiratory electron acceptor.^1,2^ Anaerobic nitrate respiratory growth is potentially hazardous. While nitrite, the initial product of reduction, may itself be toxic,^3^ its further reduction by nitrate reductase (NarG and probably also NarZ) results in cytosolic nitric oxide (NO), the major causative agent of nitrosative stress.^2,4−6^

Bacteria experience nitrosative stress when the cytotoxic radical NO and other reactive nitrogen oxides impair the function of crucial cellular components. NO reacts with other radicals, such as superoxide (O_2_^–^) and transition metal ions (principally iron), to generate reactive nitrogen species (RNS), including peroxynitrite (ONOO^–^), resulting in protein-bound iron-nitrosyls (R-Fe(NO) and R-Fe(NO)_2_) and S-nitrosothiols (RS-NO).^7^ These modifications often result in loss of function of the affected proteins. Hence, NO production is used by the innate immune system to control bacterial infections, but as noted above, some bacteria are exposed to endogenous nitrosative stress, resulting from NO generated when nitrite accumulates during anaerobic nitrate respiration.

To mitigate the deleterious effects of NO, commensal and pathogenic members of Enterobacteriaceae mount a complex and multifaceted response that is coordinated by the NO-sensitive repressor NsrR, a member of the Rrf2 protein superfamily.^8^ The intrinsic reactivity of iron–sulfur (Fe-S) clusters toward NO, while broadly deleterious, has been exploited through the evolutionary process to yield Fe-S proteins, like NsrR, that function as sensor-regulators.^9−11^ Intact [4Fe-4S] NsrR represses transcription in the absence of NO.^12^ Transcriptomic analyses of the NsrR regulon have, in most cases, consistently identified three genes (*hmp*, *hcp*, and *ytfE*) that are strongly induced by nitrosative stress.^13−16^ The Hmp protein is a flavohemoglobin oxygenase that converts NO to nitrate under aerobic conditions.^17^ The Hcp protein appears to have a dual protective role: it detoxifies NO by acting as a high-affinity anaerobic NO reductase that converts NO to nitrous oxide (N_2_O)^18^ but also appears to promote the S-nitrosylation of specific proteins as part of an additional nitrosative stress response.^19,20^

YtfE is found in many bacteria, where it contributes to protection against nitrosative stress and to survival within host tissues.^21,22^ It has been proposed that *Escherichia coli* YtfE alleviates the deleterious effects of NO-induced stress by facilitating the mobilization of Fe ions for repairing the NO-damaged Fe-S clusters of dehydratases, such as aconitase, fumarase, and dihydroxy-acid dehydratase.^16,23−26^ However, reanalysis of the *in vivo* function of YtfE, prompted by the discovery of a spontaneous 126-gene deletion in the original *ytfE* knockout mutants,^24^ revealed a YtfE-dependent decrease in aconitase and fumarase activities, a YtfE-dependent increase in cytoplasmic NO, and derepression of [4Fe-4S] NsrR-controlled promoters.^24,27^ Hence, YtfE appeared to generate NO, which was detected by NsrR and reduced to N_2_O by the high-affinity NO reductase Hcp.^27−29^

**Figure 1:**
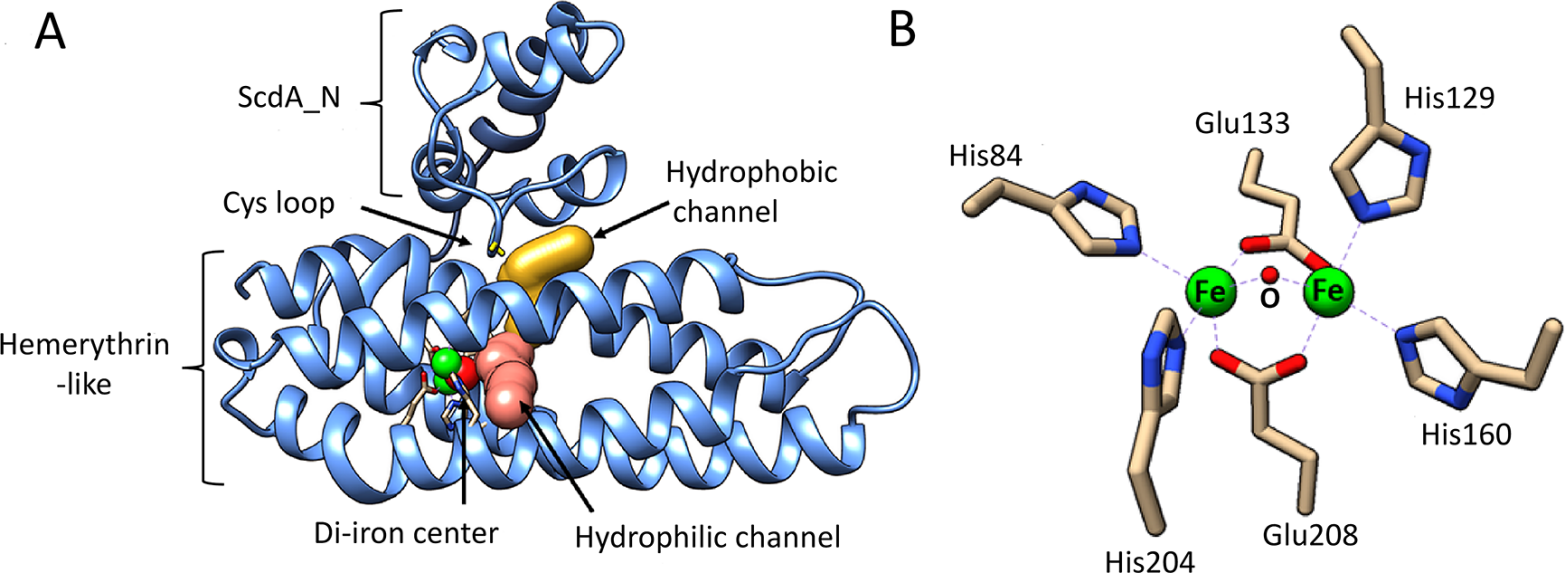
Crystal structure of YtfE. (a) Annotated structure of C30A/C31A YtfE (PDB: 5FNN). Hydrophilic (pink) and hydrophobic (yellow) channels leading to the di-iron center are shown. (b) Detailed view of the di-iron center. Molecular graphic images were made with USCF Chimera.^34^ Channels were calculated with Mole 2.0.^26,35^

YtfE is a monomeric L-shaped molecule consisting of two domains (Figure 1A). The C-terminal hemerythrin-like domain (Pfam: PF01814) contains a nonheme di-iron center.^26,30−32^ The globular N-terminal domain (domain of unknown function, DUF542, ScdA_N) contains a pair of highly conserved cysteine residues (Cys30 and Cys31) and caps a long hydrophobic channel, the length of which (∼10–25 Å) is altered by the relative position of ScdA_N to the hemerythrin-like domain.^32,33^ The thiolates of the cysteine pair are orientated toward this channel and the di-iron site and are prone to oxidation, resulting in a disulfide bond.^26,32^ A second channel, predominantly hydrophilic in nature, connects the di-iron center to the surrounding solvent.^26,32^

In comparison to the typical right-handed four α-helix bundles of hemerythrins, the hemerythrin-like domain of YtfE displays a distinctive left-handed twist.^36^ The di-iron center of YtfE is also unusual in that there are no hydrogen bonding groups or water molecules located in the vicinity of the irons. The symmetrically ligated di-iron site consists of two five-coordinate iron atoms. The coordinating ligands consist of a bridging oxygen atom, two bridging carboxylates (from Glu133 to Glu208), and two pairs of ε-nitrogen atoms from histidine residues (His84, His204 and His129, and His160), resulting in a distorted octahedral geometry, with vacant positions on each iron opposite to the bridging oxygen (Figure 1B).^31,32,36^ In aerobically isolated YtfE, the di-iron site is in an EPR-active (*g* = 1.96, 1.92, and 1.88), mixed-valent Fe^2+^/Fe^3+^ state (semi-met form according to the hemerythrin literature^37^). Oxidation or reduction results in the di-ferric Fe^3+^/Fe^3+^ (met) or di-ferrous Fe^2+^/Fe^2+^ (deoxy) states, respectively, both of which are EPR-silent^30^ and retain the bridging oxygen.^32^

*In vitro*, NO readily binds to the di-ferrous di-iron site of YtfE and its orthologues (e.g., *Ralstonia eutropha* NorA), yielding iron-nitrosyls. Nitrosylated NorA displays an EPR spectrum centered on *g* = 2.03, indicative of a dinitrosyl iron complex (DNIC), while YtfE displays an EPR signal centered on *g* = 3.95, indicative of one or more *S* = 3/2 mononitrosyl iron complexes (MNICs).^32,38,39^ It has also been reported that YtfE and NorA are capable of slowly reducing NO or nitrite to N_2_O or NO, respectively.^32,38,39^ These observations have led to various proposals for the roles of YtfE and homologues, including NO detoxification through sequestration (resulting in DNIC species)^39^ and as a NO reductase (generating N_2_O).^32^

Here, we used spectroscopic, mass spectrometric, and kinetic approaches to re-evaluate the properties of *E. coli* YtfE. We demonstrate that it belongs to a new class of nitrite reductases, which efficiently generates and releases NO, and that YtfE can only support the repair of Fe-S clusters under conditions that degrade its di-iron center. These data are discussed in the context of extensive *in vivo* data, clarifying our understanding of the role of YtfE in *E. coli*, which likely extends to the roles of YtfE orthologues in other bacteria.

## Results and Discussion

### Effects of Nitrite and NO on *E. coli* Strains Sensitive to Nitrosative Stress

Balasiny *et al*.^27^ demonstrated a YtfE-dependent accumulation of NO in the cytoplasm of bacteria defective in previously characterized nitrite reductases (encoded by *nirBD* and *nrfAB)* and NO-detoxifying enzymes (*norVW*, *hcp*, and *hmp*). Rather than providing iron to repair metallo-proteins inactivated by NO, it was suggested that YtfE is an enzyme that releases NO, either directly or indirectly, into the cytoplasm.^27,28^ We note that the *R. eutropha* YtfE orthologue NorA has been reported to reduce nitrite to NO *in vitro.*(38) Thus, YtfE may function as a NO-generating nitrite reductase. *In vivo* experiments were conducted to test this possibility*.*

**Figure 2:**
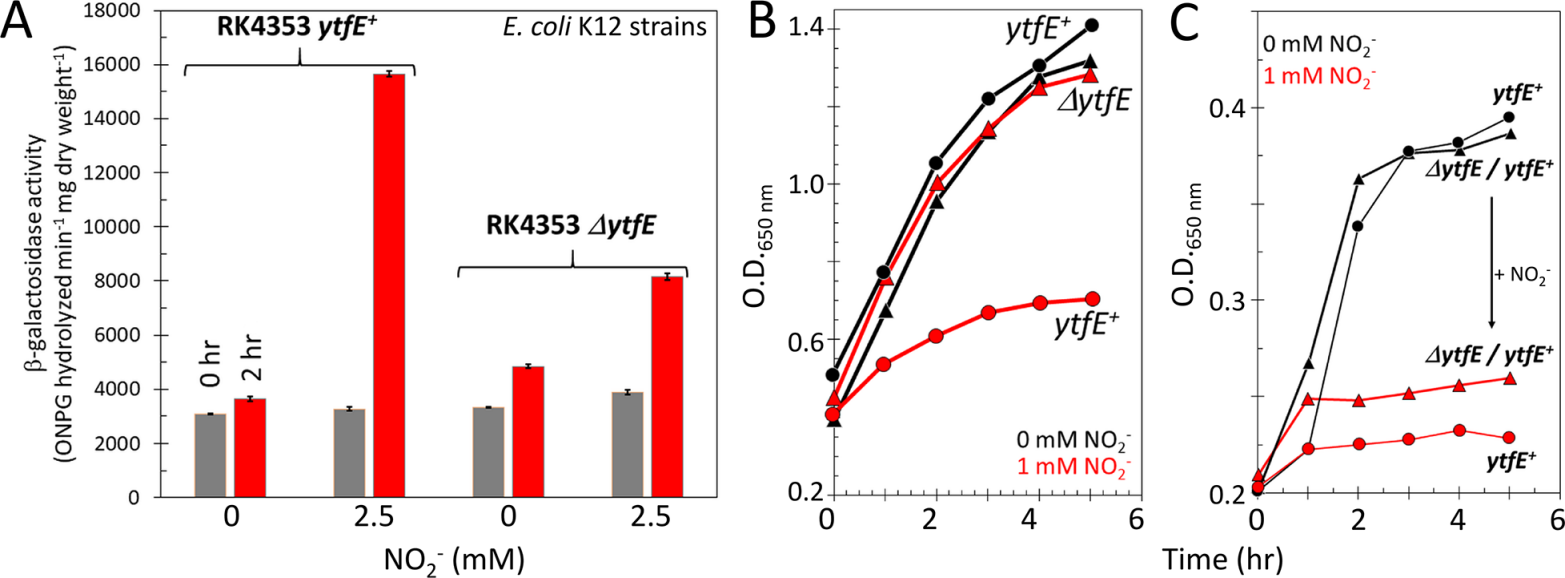
Effect of nitrite (NO_2_^–^) on strains sensitive to nitrosative stress. (A) Strain RK4353 (*ytfE*^+^) or JCB5211 (RK4353 Δ*ytfE*) was transformed with the reporter plasmid pNF383 and grown in duplicate anaerobic cultures. When OD_650 nm_ reached 0.2, one culture was treated with 2.5 mM NaNO_2_, and the other served as a control. Samples were assayed for β-galactosidase activity at the times indicated. β-Galactosidase activity was significantly (*p* = 0.0062) higher in response to nitrite in RK4353 (*ytfE*^+^) than JCB5211 (RK4353 Δ*ytfE*). Error bars indicate standard deviation (*n* = 4) from biological replicates. (B) Strains JC5280 (Δ*ytfE*) and JCB5270 (*ytfE*^+^), which both lack nitrate and nitrite reductases and NO-consuming enzymes, were grown in the presence (red line) or absence (black line) of 1 mM NaNO_2_ and growth was monitored (OD_650 nm_) for ∼6 h post addition. (C) Strain JC5280 (Δ*ytfE*) (triangles) or JC5270 (*ytfE^+^*) (circles) transformed with pBB2016, a *ytfE* expression plasmid, in the presence (red line) or absence (black line) of 1 mM NaNO_2_. The OD_650 nm_ of samples removed at intervals was determined. The arrow indicates nitrite-induced change in growth. Growth curves were repeated at least twice on different days with different inoculum and medium batches. Representative results from single experiments are shown.

*E. coli* strain RK4353 (laboratory wild-type strain, *ytfE*^+^) or JCB5211 (RK4353 Δ*ytfE*, see Methods) was transformed with pNF383, a reporter plasmid that features β-galactosidase (*lacZ*) expression under the control of the *hcp* promoter. The *hcp* promoter is subject to transcriptional repression by NsrR,^6^ and thus, β-galactosidase activities report on cytosolic NO levels. The transcription of *lacZ* was significantly (*p* = 0.0062) higher in response to nitrite in the parental strain than in the Δ*ytfE* mutant (Figure 2A), consistent with YtfE-dependent cytoplasmic NO production from nitrite.

For subsequent experiments, we used JCB5270 (*ytfE*^+^) and JCB5280 (Δ*ytfE*) that lack nitrate (*narGHJI* and *narZ*) and nitrite (*nirBD* and *nrfAB*) reductases and NO-consuming enzymes (*norVW*, *hcp*, and *hmp*).^27^ Under anaerobic conditions, the Δ*ytfE* strain (JCB5280) grew similarly in the presence or absence of 1 mM nitrite (Figure 2B). In contrast, the *ytfE^+^* strain (JCB5270) was severely inhibited by the presence of 1 mM nitrite (Figure 2B). To assess whether the different effects of nitrite on growth were due solely to the presence or absence of *ytfE*, JCB5270 (*ytfE*^+^) and JCB5280 (Δ*ytfE*) were transformed with a low copy number plasmid (pBB2016) expressing *ytfE* under the control of its own promoter. The transformed strains were able to grow in the absence of nitrite but were severely inhibited by 1 mM nitrite (Figure 2C). Taken together, these experiments strongly suggest that the physiological role of YtfE is to reduce nitrite to NO, which, in the absence of the NO-consuming enzymes NorVW, Hcp, NrfA, and Hmp, resulted in impaired growth.

### Characterization and Redox Cycling of Anaerobically Purified YtfE

Several studies have shown that *ytfE* is expressed *in vivo* under microaerobic or anaerobic conditions in response to the presence of nitrate, nitrite, or nitrosative stress.^16,23,24,30,40^ As the di-iron center is stable in the presence of air, aerobic conditions have been used for most purifications to generate YtfE in mixed-valent (Fe^3+^/Fe^2+^) and/or di-ferric states. This purification strategy also leads to the formation of intramolecular (between Cys30 and Cys31) and intermolecular disulfide bonds, the latter resulting in YtfE dimers.^23,32,38^ Therefore, we chose to purify YtfE under anaerobic conditions.

**Figure 3:**
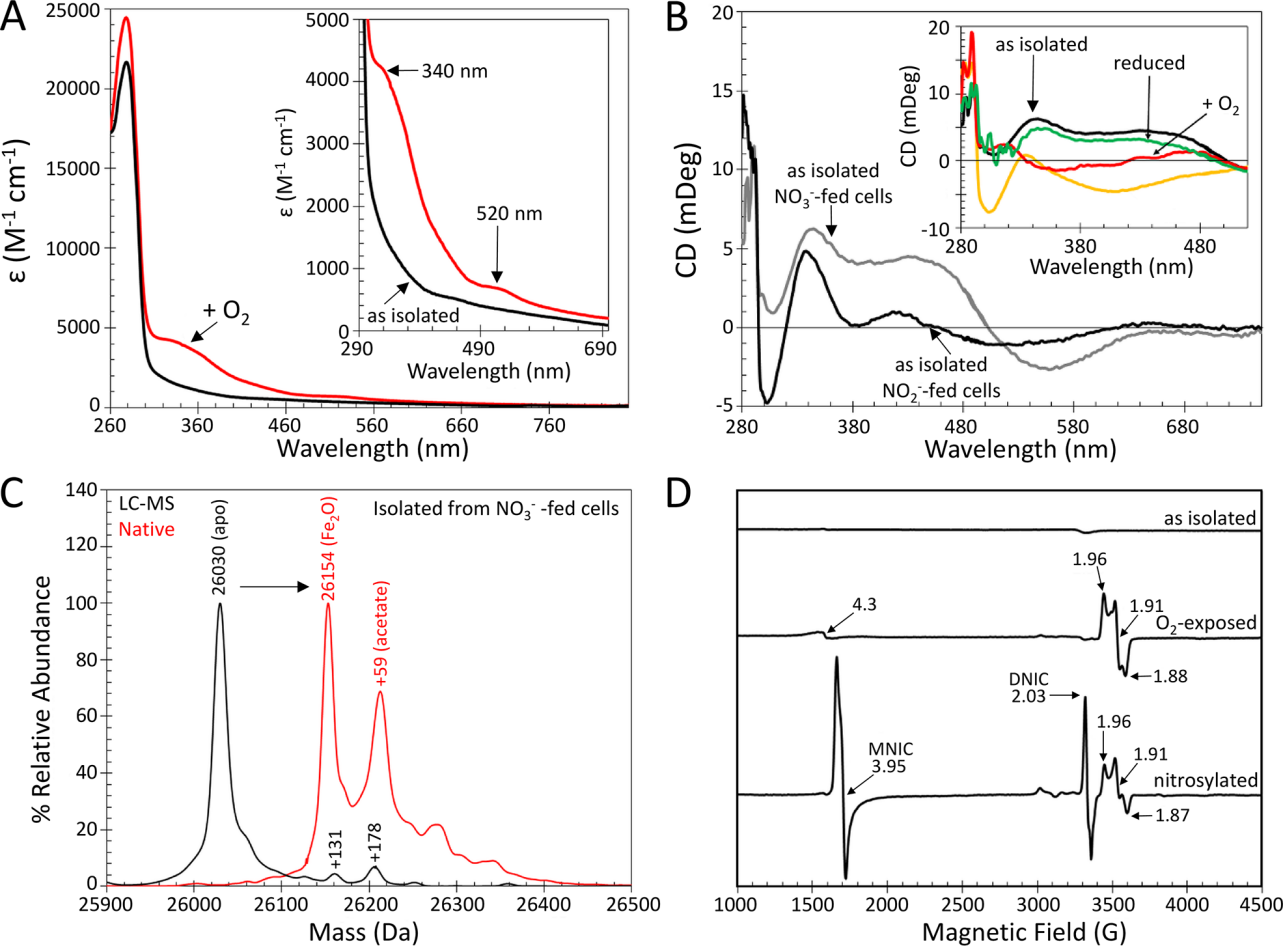
Spectroscopic characterization of YtfE. (A) Absorbance spectra of as-isolated (black line) and air-exposed (red line) YtfE. (B) CD spectra of as-isolated YtfE from nitrate-supplemented (NO_3_^–^; gray line) and nitrite-supplemented (NO_2_^–^; black line) cultures (BL21 ΔDE3 Δ*fnr*); inset: deoxy YtfE exposed to air (red line), dithionite-reduced (green line), and nitrosylated (yellow line). (C) Positive mode denaturing LC–MS (black line) and native mass spectrometry (red line) of as-isolated YtfE from nitrate-supplemented cultures. Under native conditions, higher mass species were detected, corresponding to YtfE containing its di-iron cofactor and an acetate adduct of the cofactor-bound form, as indicated. (D) EPR spectra of YtfE as isolated and following exposure to O_2_ and NO (nitrosylated), as indicated. Spin quantification indicated that the mixed-valent species accounted for ∼40 and ∼20% of YtfE following exposure to O_2_ and NO, respectively, and MNIC and DNIC species represented ∼60 and ∼10% of YtfE concentration following NO exposure. The spectra were recorded with the following parameters: temperature, 10 K; microwave frequency, 9.424 GHz; microwave power, 6.3 mW; modulation frequency, 100 kHz; modulation amplitude, 5 G.

Anaerobically prepared YtfE from nitrate-supplemented cultures was investigated using absorbance, CD and EPR spectroscopies, native (nondenaturing) mass spectrometry, and ICP-MS. For a full description of the data summarized below, see the Supporting Information. As-isolated YtfE was largely monomeric and contained two Fe per protein, and the di-iron site was in the reduced (Fe^2+^/Fe^2+^) form. Limited exposure of as-isolated di-ferrous YtfE to air resulted in oxidation of the di-iron site, with ∼40% in the mixed-valent (Fe^3+^/Fe^2+^) form, as determined by EPR spin quantification, and the remaining ∼60% present as di-ferric (Fe^3+^/Fe^3+^) YtfE, with some possibly in the O_2_-bound form (Figure 3, Figures S1–S3, and Tables S1 and S2). Anaerobic addition of dithionite or DTT to air-exposed, oxidized YtfE resulted in re-reduction to di-ferrous YtfE, demonstrating the ability of the cofactor to undergo redox cycling with no significant loss of iron (Figure 3B, inset, and Figure S3). The dependence of the rate of reduction on DTT concentration was consistent with a relatively weak interaction between DTT and YtfE (Figure S4). Ascorbate and NADH were less efficient reductants of oxidized YtfE, while glutathione was completely ineffective (Figure S3B,C). The data suggest that the accessibility of the reductant to the protein/di-iron center (and not just reduction potential) is important for reduction to occur.

### Interaction of YtfE with Nitrite and NO

*E. coli* BL21 (DE3), commonly used for protein expression, is an *fnr* mutant and is thus defective in anaerobic nitrate/nitrite respiration due to impaired expression of genes coding for nitrate/nitrite reductases, NarG, NapA, NrfA, or NirB.^41−43^ When YtfE (expressed from pGS2618) was isolated from *E. coli* BL21 (DE3) cultures supplemented with nitrite (instead of nitrate, see Methods), it was pale yellow when concentrated and gave a CD spectrum distinct from that of YtfE isolated from nitrate-supplemented cells, with features at (+)340, 420, and 540 nm and (−)305 nm, suggesting the presence of a mixture of redox states (Figure 3B). To provide further insight into these states, di-ferrous YtfE was exposed to NO, resulting in a nitrosylated form with CD features at (+)335 nm and (−)305 and 400 nm (Figure 3B, inset, yellow trace). The presence of a (−)305 nm band in the YtfE sample above suggested the presence of nitrosylated YtfE in nitrite-supplemented cultures.

The deconvoluted native mass spectrum for monomeric YtfE isolated anaerobically from nitrite-supplemented cells and ionized from ammonium acetate, like YtfE from nitrate-supplemented cells, displayed two major peaks but was otherwise distinct. The peak at 26,152 Da corresponded to the di-iron-bound form (Figure S1E and Table S1), while that at 26,206 Da indicated a mixture of species that could be resolved by peak fitting as two separate species at 26,199 and 26,211 Da, corresponding to the addition of nitrite (+46 Da) and acetate (+59), respectively (Figure S1E, inset). Lower mass peaks at 26,084 and 26,114 Da corresponded to YtfE containing a single iron atom and a possible Fe-NO species, respectively (Figure S1E).

The detection by mass spectrometry of acetate, formate, carbonate, and nitrite adducts of YtfE (Figure 3C and Figure S1B,C) suggests that the natural substrate might contain an O=*X*—O^–^ functional group. We note that a variant of bacteriohemerythrin from *Methylococcus capsulatus* (Bath)*,* which contains a di-iron site with improved solvent accessibility, has been crystallized with a nitrate/acetate anion bound to the di-iron site.^44^

### Spontaneous Reduction of Nitrite to NO by Di-Ferrous YtfE

**Figure 4:**
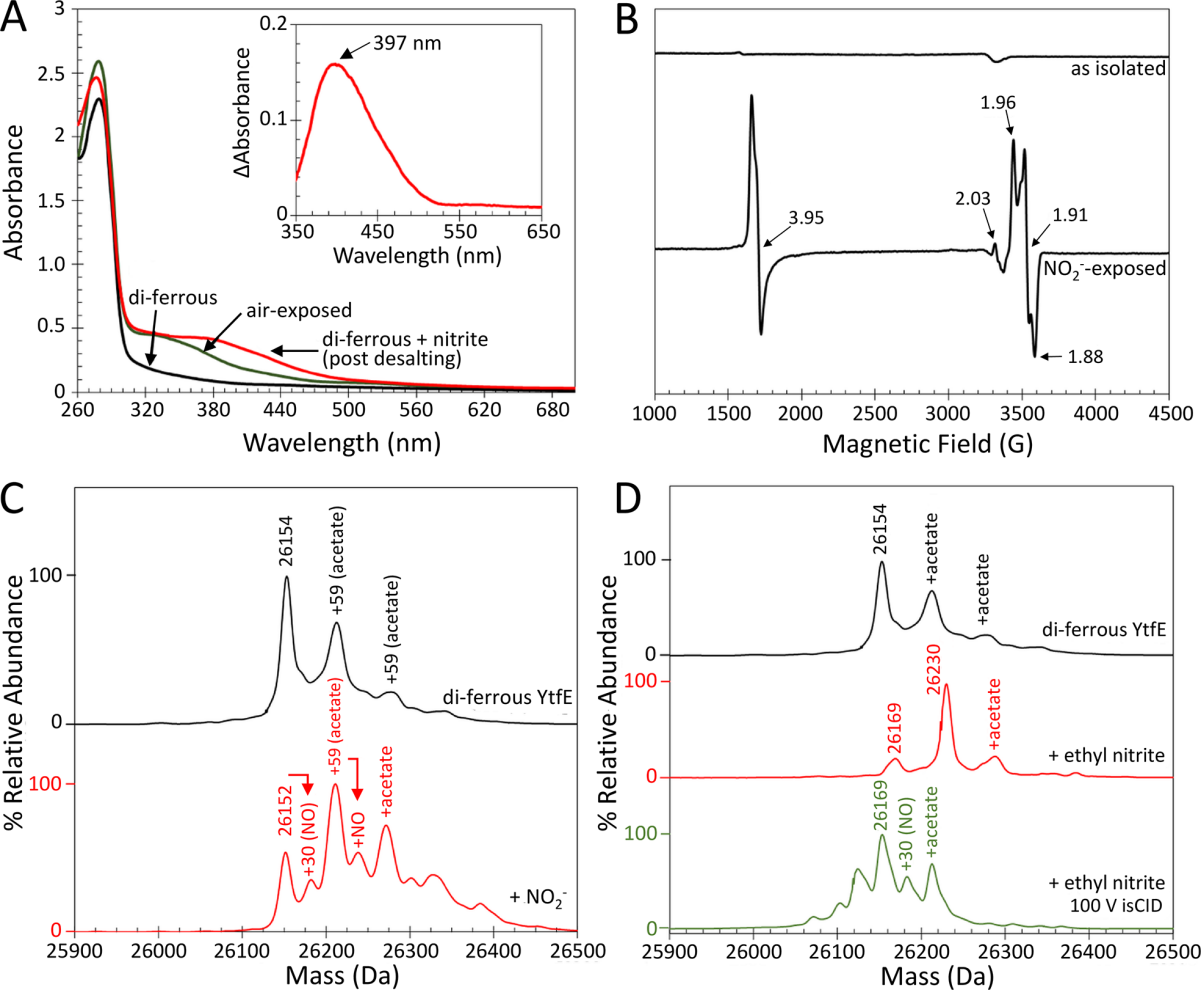
Autonitrosylation of di-ferrous YtfE by nitrite. (A) UV–visible absorbance spectra of di-ferrous YtfE (black line) and nitrite-treated di-ferrous YtfE (red line) post desalting. Air-exposed (mixed-valent/di-ferric) YtfE is shown for comparison (green line). Inset: the difference spectrum between mixed-valent and nitrite-treated YtfE reveals a band at 397 nm, indicative of iron-nitrosyl species. (B) EPR spectra of as-isolated di-ferrous YtfE, pre- and post-treatment with nitrite. Signals arising from MNIC (*g* = 3.95), DNIC (*g* = 2.03), and mixed-valent YtfE (*g* = 1.96, 1.91, and 1.88) are indicated. Note that the EPR spectrum of air-exposed YtfE is shown in Figure 3D. The native mass spectrometry of di-ferrous YtfE treated with (C) nitrite or (D) ethyl nitrite. Nitrosylated YtfE is observed after nitrite, but not ethyl nitrite, treatment. An ethyl nitrite adduct (26,230 Da) was the major species in the latter. A minor peak due to an acetate adduct of the ethyl nitrite adduct was also observed at 26,289 Da. On the low mass side, an oxygen atom (+16 Da) adduct of YtfE was observed at 26,169 Da. The lower spectrum in panel (D) was obtained through in-source collision (isCID), which resulted in the breakdown of ethyl nitrite to give the nitrosylated adduct, along with some unknown lower mass species.

The observation of a likely nitrite-bound form of YtfE from nitrite-supplemented cultures prompted us to investigate the effect of nitrite on YtfE *in vitro*. Addition of KNO_2_ (3 mM) to di-ferrous YtfE (102 μM) resulted in the immediate appearance of a yellow color. Small molecules (≤5 kDa) were subsequently removed via a desalting column and a UV–visible spectrum recorded, revealing a new feature extending out to ∼500 nm (Figure 4A). A difference spectrum, generated by subtracting the oxidized (air-exposed) spectrum from the nitrite-treated spectrum, revealed a prominent peak centered at 397 nm, indicative of iron-nitrosyl complexes (Figure 4A, inset).^38,39^ Control reactions with DTT, ferrous ammonium sulfate, and nitrite, or DTT and nitrite, did not generate a yellow compound upon mixing.

The yellow form of YtfE was further characterized by EPR and native mass spectrometry. The EPR spectrum of nitrite-treated YtfE displayed a major signal centered on *g* = 3.95 and a minor signal at *g* = 2.03, characteristic of MNIC and DNIC species, respectively,^32,39,45^ in addition to mixed-valent YtfE (*g* = 1.96, 1.91, and 1.88) (Figure 4B).^30^ The intensity of the DNIC signal was significantly less than that observed in di-ferrous YtfE exposed to excess NO (Figure 3D and Figure S2B). Spin quantification indicated that the MNIC and mixed-valent forms were both at ∼35% of the YtfE concentration. The deconvoluted native mass spectrum of nitrite-treated YtfE ionized from ammonium acetate buffer contained several peaks. The first, at 26,152 Da, corresponded to either di-ferrous YtfE with an intramolecular disulfide bond or mixed-valent/di-ferric YtfE (Figure 4C and Table S1). A lower intensity peak, at 26,183 Da, corresponded to the addition of a single NO to mixed-valent/di-ferric YtfE. Further peaks due to one and two acetate adducts (+59 and +118 Da) were also observed, as was an acetate/NO adduct (+89 Da; Figure 4C).

Ethyl nitrite, an alkyl analogue of nitrite, has been used previously to study the interaction of hemerythrin with nitrite. The addition of ethyl nitrite (1.5 mM) to di-ferrous YtfE resulted in a prominent peak at 26,229 Da, corresponding to an ethyl nitrite adduct (+75 Da) (Figure 4D). As there was no evidence for the turnover of ethyl nitrite, in-source collision-induced dissociation (isCID) was used to “activate” the YtfE-ethyl nitrite species. Application of 100 V isCID resulted in a more complex spectrum with a prominent peak at 26,154 Da corresponding to di-ferrous YtfE and additional peaks on the high mass side at 26,184 and 26,213 Da due to mononitrosylated YtfE (+30 Da) and an acetate adduct (+59 Da) of YtfE, respectively (Figure 4D).

The observation of mononitrosylated YtfE via mass spectrometry is consistent with the EPR observations reported here and previously.^32^ It also suggests that di-ferrous YtfE, like hemerythrin, can facilitate the one-electron reduction of nitrite to NO.^46,47^

### Kinetics of YtfE-Catalyzed Nitrite Reduction

In the presence of ascorbate (+60 mV versus standard hydrogen electrode (SHE) at pH 7.0) and phenazine methosulfate (+80 mV),^48^ the *R. eutropha* YtfE orthologue NorA was able to catalyze a limited number of nitrite reductions.^38,39^ The reaction was slow; the *K*_m_ (∼7 mM) for nitrite was high and was subject to inhibition by NO. In contrast, *E. coli* YtfE has been reported to reduce NO to N_2_O in the presence of ascorbate and *N*,*N*,*N*′,*N*′-tetramethyl-*p*-phenylenediamine (+276 mV), or NADH (−320 mV), albeit rather slowly.^32,33^ While ascorbate and NADH can clearly act as reductants for YtfE, they are less efficient than DTT (Figure S3B,C). Hence, the reduction of YtfE by ascorbate or NADH may be the rate-limiting step in these previously reported assays.

We note that the potentials of mixed-valent/di-ferrous and di-ferric/mixed-valent redox couples of YtfE are +110 and +260 mV, respectively, and that the standard reduction potential for the NO_2_^–^/NO couple is +375 mV at pH 7.0,^30,49^ consistent with the proposal that nitrite might be a physiological substrate for YtfE. Efficient reduction of YtfE by DTT (−330 mV) suggests that YtfE might require a reductant with better access to the protein than ascorbate or NADH. The need for an external reductant opens the possibility that YtfE might interact with, for example, a ferredoxin and associated reductase. Reduction potentials for ferredoxin [4Fe-4S]^2+/1+^ clusters typically fall between −300 and −700 mV and are highly dependent upon the protein scaffold.^50−52^

**Figure 5:**
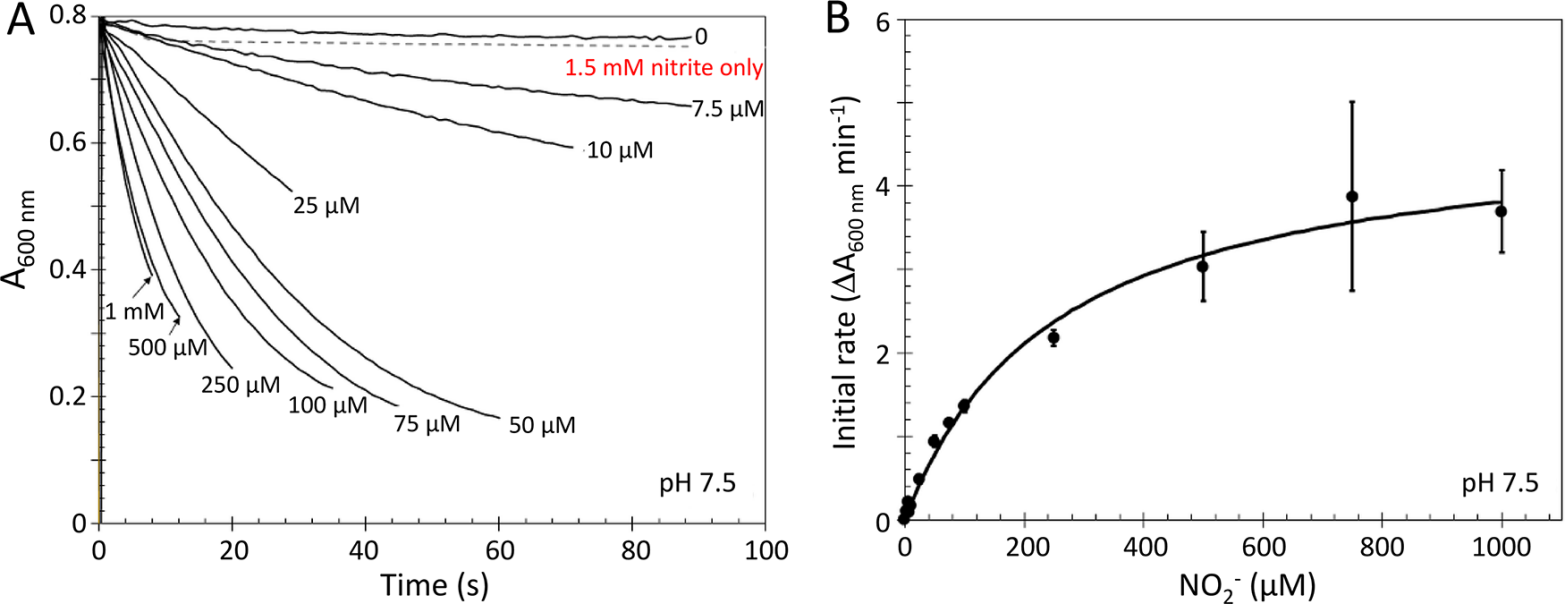
Kinetics of nitrite reduction. (A) YtfE-mediated oxidation of methyl viologen in response to increasing concentrations of nitrite. Note that data for 750 μM were omitted for clarity. (B) Initial rate analyses of data in panel (A). Fitting to a Michaelis–Menten equation (black line) gave a *K*_m_ of ∼250 μM for nitrite. See Table 1 for full kinetic parameters. Error bars indicate ± standard deviation.

To mimic the action of low potential [4Fe-4S] ferredoxins, methyl viologen (−450 mV), which has been used in nitrate and nitrite reduction assays as a substitute for natural redox partners, was used to assess the nitrite reductase activity of YtfE over a physiological pH range.^53^ Following the addition of di-ferrous YtfE (10 μM), a decrease in *A*_600 nm_ was observed due to methyl viologen (58 μM) oxidation, the rate of which was dependent upon the concentration of nitrite and the presence of YtfE (Figure 5A). Under conditions where reductant was in excess, quantification of the oxidation of methyl viologen indicated an ∼1:1 ratio between nitrite and reductant consumed, consistent with a one-electron reduction of nitrite to NO. A plot of the initial rate (Δ*A*_600 nm_ min^–1^) against nitrite concentration yielded a Michaelis–Menten saturation curve typical of an enzyme reaction (Figure 5B). Fitting of the data gave a *K*_m_ of ∼250 μM and a *k*_cat_ of ∼35 min^–1^ at pH 7.5 (see Table 1 for other kinetic parameters).

**Table 1:** Kinetic Parameters for Nitrite Reduction Catalyzed by YtfE

mediator	potential (mV) versus SHE	pH	YtfE^a^	*K*_m_ (μM NO_2_^–^)	*V*_max_^b^ (μmol min^–1^ mg^–1^)	*k*_cat_ (min^–1^)	*k*_cat_/*K*_m_ (M^–1^ min^–1^)
Fe^2+^(*RS*)_*n*_	–330	7.5	wt	88 (± 13)	0.04 (± 0.001)	0.97 (± 0.04)	1.10 (± 0.044) × 10^4^
		7.5	wt + O_2_	242 (± 81)	0.02 (± 0.003)	0.56 (± 0.07)	0.23 (± 0.028) × 10^4^
		7.5	C30A/C31A	n.d.	∼0.001	∼0.03	n.d.
		7.5	CAM-wt	n.d.	∼0.002	∼0.05	n.d.
							
safranin O	–289	7.5	wt	252 (± 32)	0.22 (± 0.01)	5.74 (± 0.20)	2.28 (± 0.080) × 10^4^
		7.5	C30A/C31A	251 (±32)	0.23 (± 0.01)	6.11 (± 0.30)	2.43 (± 0.119) × 10^4^
		7.5	CAM-wt	252 (± 32)	0.23 (± 0.01)	6.12 (± 0.38)	2.43 (± 0.150) × 10^4^
							
methyl viologen	–450	7.0	wt	279 (± 70)	2.40 (± 0.23)	62.7 (± 5.95)	22.49 (± 2.132) × 10^4^
		7.5	wt	252 (± 32)	1.33 (± 0.01)	34.8 (± 1.59)	13.81 (± 0.631) × 10^4^
		7.5	C30A/C31A	288 (± 29)	1.59 (± 0.01)	41.6 (± 1.58)	14.44 (± 0.550) × 10^4^
		8.0	wt	246 (± 32)	0.76 (± 0.03)	19.8 (± 0.82)	8.04 (± 0.333) × 10^4^

a Note that wt is wild-type YtfE and CAM-wt is carboxymethylated YtfE.

b Mediator-dependent data, expressed as NO-generated (μmol min^–1^ mg^–1^) (YtfE), safranin O-oxidized (μmol min^–1^ mg^–1^), or methyl viologen-oxidized (μmol min^–1^ mg^–1^), respectively.

The nitrite reductase assays were repeated with dithionite-reduced safranin O (−289 mV) in place of methyl viologen to mimic less reducing [2Fe-2S]^2+/1+^ ferredoxins.^52^ The reaction remained nitrite-dependent, with a similar *K*_m_ but a lower *k*_cat_, indicating less efficient catalysis in the presence of safranin O (Figure S5E and Table 1). Control reactions lacking YtfE, but containing 1.5 mM KNO_2_, did not result in the oxidation of methyl viologen or safranin O over the same time frame as YtfE-containing assays. Nitrate failed to elicit a reaction in the presence or absence of YtfE.

Over a physiologically relevant pH range (pH 7.0–8.0), the rate of the methyl viologen-mediated reaction was clearly pH-dependent, decreasing linearly with decreasing [H^+^] (Figure S6). This could indicate that the oxidant might be nitrous acid (HNO_2_) rather than nitrite.^46^ We note that evidence from resonance Raman experiments using H_2_^18^O indicated that the YtfE μ-oxo-bridge is solvent-derived and that no spectral changes were observed in the presence of D_2_^16^O, ruling out the involvement of a preformed bridging hydroxyl prior to the binding of nitrite.^31^ We also note that the reduction of nitrite by hemerythrin has been proposed to proceed via HNO_2_ in an “inner sphere” process.^46^ The increased membrane permeability of HNO_2_ would likely more than compensate for its low abundance at physiological pH (∼6 nM, pH 7.5), and once across the membrane, the [HNO_2_]:[NO_2_^–^] equilibrium was re-established.^54^ However, the observed pH dependence could also be due to the requirement for a proton to complete the catalytic cycle (Figure 7) or the acid–base behavior of residue side chains near the active site. We note that the structure of YtfE does not feature water molecules or H-bonding side chains near the di-iron active site.^32^

**Figure 6:**
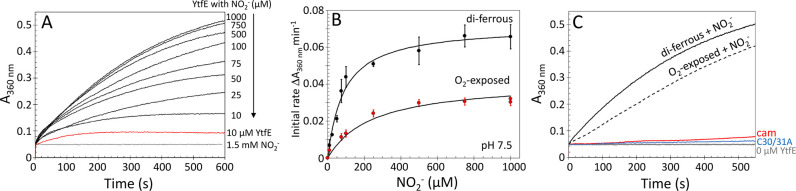
Kinetics of YtfE-dependent formation of iron-nitrosyl species. (A) Di-ferrous YtfE-mediated formation of iron-nitrosyl species (measured at 360 nm) in response to increasing concentrations of nitrite. (B) Initial rate analysis of the data (black circles) in comparison to equivalent experiments performed with air-exposed (oxidized) YtfE (red circles). Fitting to a simple Michaelis–Menten equation (black lines) gave *K*_m_ values for nitrite of 88 and 242 μM for di-ferrous and mixed-valent YtfE, respectively. Error bars indicate standard deviation (*n* ≥ 4). (C) Plots of *A*_360 nm_ showing the effect of Cys30Ala/Cys31Ala YtfE (blue line) and carboxymethylation (red line). The responses of di-ferrous and mixed-valent YtfE in the presence of 1.5 mM KNO_2_ are shown for comparison. See Table 1 for full kinetic parameters.

Iron-nitrosyl complexes are readily formed *in vitro* and *in vivo* from NO, a suitable ligand, and free hexa-aqua iron.^55−58^ In the presence of glutathione, iron-nitrosyl complexes display intense absorption bands between 300 and 400 nm^59^ and might form by sequestering free NO in the solution, potentially preventing product inhibition of YtfE under assay conditions. The addition of di-ferrous YtfE to increasing amounts of nitrite (up to 1.5 mM) in the presence of excess (3 mM) glutathione and iron (200 μM Fe^2+^) resulted in the formation of a new species with absorbance properties (λ_max_ = 360 nm), indicative of Roussin’s red ester (RRE)-type glutathione iron-nitrosyl complex, [Fe_2_(NO)_4_(GSH)_2_].^59^ Control reactions lacking YtfE failed to produce iron-nitrosyl complexes over the same time frame. Thus, here, Fe^2+^, present as Fe(*RS*)_*n*_ (−330 mV^60^), acts as an effective reductant to support catalytic turnover. The increase in absorbance at 360 nm was used to detect *in situ* iron-nitrosyl formation and track the kinetics of the reaction (Figure 6A). At high nitrite concentrations, effectively all the added iron was in the form of RRE, indicating that Fe^2+^ (the reductant) was limiting under these conditions. A plot of the initial rate (Δ*A*_360 nm_ min^–1^) against nitrite concentration again yielded a Michaelis–Menten saturation curve typical of an enzyme-catalyzed reaction (Figure 6B), yielding a *K*_m_ of ∼90 μM and a *k*_cat_ of ∼1 min^–1^ at pH 7.5 for nitrite reduction (Table 1). Although this nitrite reductase activity is lower than that measured with alternative reductants, the *K*_m_ for nitrite was lower and within the expected intracellular concentration range (≤0.1 mM) for nitrite in *E. coli*. The use of air-exposed YtfE resulted in an apparently impaired ability to produce iron-nitrosyls, with a *K*_m_ of ∼240 μM and *k*_cat_ of ∼0.6 min^–1^ (Figure 6B and Table 1).

### YtfE Is Not an Efficient NO Reductase

The previous proposal that YtfE functions as a NO reductase^32^ was based on detection of the relatively slow YtfE-catalyzed reduction of NO to N_2_O.^32,33^ To address the possibility that the NO generated by nitrite reduction is an intermediate of N_2_O formation, YtfE-catalyzed N_2_O formation from nitrite was investigated using GC headspace analysis. Reaction of di-ferrous YtfE (437 μM) with nitrite (6.14 mM) and DTT (2.7 or 5.4 mM) resulted in detection after 15 min of <0.05 N_2_O per YtfE at the highest reductant concentration and ∼0.7 N_2_O per YtfE after 4 h (<0.003% of total nitrite converted to N_2_O). To directly compare N_2_O and NO formation, the GC headspace analysis of N_2_O and EPR quantification of the MNIC signal were performed following addition of nitrite (6 mM) to di-ferrous YtfE (437 μM) in the absence of a reductant (i.e., single turnover conditions). This revealed ∼0.007 N_2_O and >0.3 NO per YtfE (see the Supporting Information), consistent with the facile reduction of nitrite to NO but not the further reduction of NO to N_2_O. Overall, formation of N_2_O was very inefficient, and we conclude that YtfE is unlikely to function as a NO reductase *in vivo*.

### Cys30 and Cys31 Play a Role in Electron Transfer to the YtfE Di-Iron Center and Nitrite Reduction

Here, we have shown that di-ferrous YtfE can facilitate the one-electron reduction of nitrite to NO. The globular ScdA_N domain positions the conserved cysteine pair (Cys30 and Cys31) within 10 Å of the di-iron center via a hydrophobic channel. It has been proposed that this cysteine pair might act as relay between an unknown external electron donor and the di-iron site.^32^

Low molecular weight thiols, such as glutathione, play an important role in the maintenance of reduced protein thiols *in vivo* through disulfide exchange, and we note that Cys30/31 readily form intra- and intermolecular disulfides.^32,61^ Glutathione is found in high concentrations in the cytoplasm of many organisms, including *E. coli*, and its redox chemistry dominates the cytoplasm, maintaining a reduction potential of −260 to −290 mV under normal conditions.^62^ Although quiescent YtfE appears to be maintained in a reduced form *in vivo*, we showed above (Figure S3B) that reduced glutathione is incapable of acting as a direct reductant for the di-iron site. Although the di-iron site of di-ferrous YtfE can serve as a stoichiometric electron source for reduction of nitrite, it must be re-reduced for catalytic turnover.

Several reductants of the YtfE di-iron center that are capable of supporting catalytic nitrite reduction were identified above, including DTT, methyl viologen, and safranin O. Carboxymethylated YtfE (CAM-YtfE), generated by reaction with iodoacetamide and containing alkylated Cys residues (Figure S3D), remained susceptible to reduction by DTT, but the rate of reduction decreased by 50%, suggesting that Cys30 and Cys31 might play a role in DTT-mediated reduction (Figure S3B). However, the effect depended on which external reductant was employed because with the redox mediators methyl viologen and safranin O, no inhibitory effect was observed (Table 1). With Fe(*RS*)_*n*_ as a reductant (Figure 6), CAM-YtfE was incapable of supporting catalysis, indicating that Fe(*RS*)_*n*_-mediated reduction is strictly dependent upon Cys30/31 (Figure 6C).

Carboxymethylation of YtfE resulted, on average, in the addition of more than two carboxymethyl groups per protein, raising the possibility that the observed effects might be due to modifications other than those at Cys30/31 (YtfE contains another Cys residue, Cys 184). Thus, a C30A/C31A variant of YtfE was generated and purified. Mass spectrometry confirmed the presence of the double substitution and that the protein contained a di-iron center (Figure S5A). The di-iron center of C30A/C31A YtfE remained redox-active as judged by optical and EPR spectrometry (Figure S5B,C) but did not autonitrosylate to the same extent as YtfE upon exposure to nitrite. EPR and native mass spectrometry on comparable C30A/C31A YtfE samples confirmed the lack of autonitrosylation.

Experiments with DTT showed that the absence of the two Cys residues significantly affected the rate of reduction, with behavior similar to that of CAM-YtfE (Figure S3B). With Fe(*RS*)_*n*_ as a reductant, the absence of the Cys residues caused a more severe effect (as also observed for CAM-YtfE), again confirming the requirement of Cys30/31 for catalysis under these conditions (Figure 6C). In addition to glutathione, the cytoplasmic thiol redox system of *E. coli* comprises thioredoxin and glutaredoxin disulfide reductases that reduce disulfide bonds of multiple client proteins through thiol-disulfide exchange. The behavior of wild-type and C30A/C31A YtfE toward DTT, a small-molecule thioredoxin mimic, but not GSH, suggests that under certain conditions, Cys30/31 can reduce the di-iron center through disulfide bond formation. No effect on catalysis was observed when methyl viologen and safranin O were used as reductants, indicating that they supply electrons via a different mechanism, which does not involve Cys30/31 (Figure S5E).

### Mechanism of YtfE-Catalyzed Nitrite Reduction

**Figure 7:**
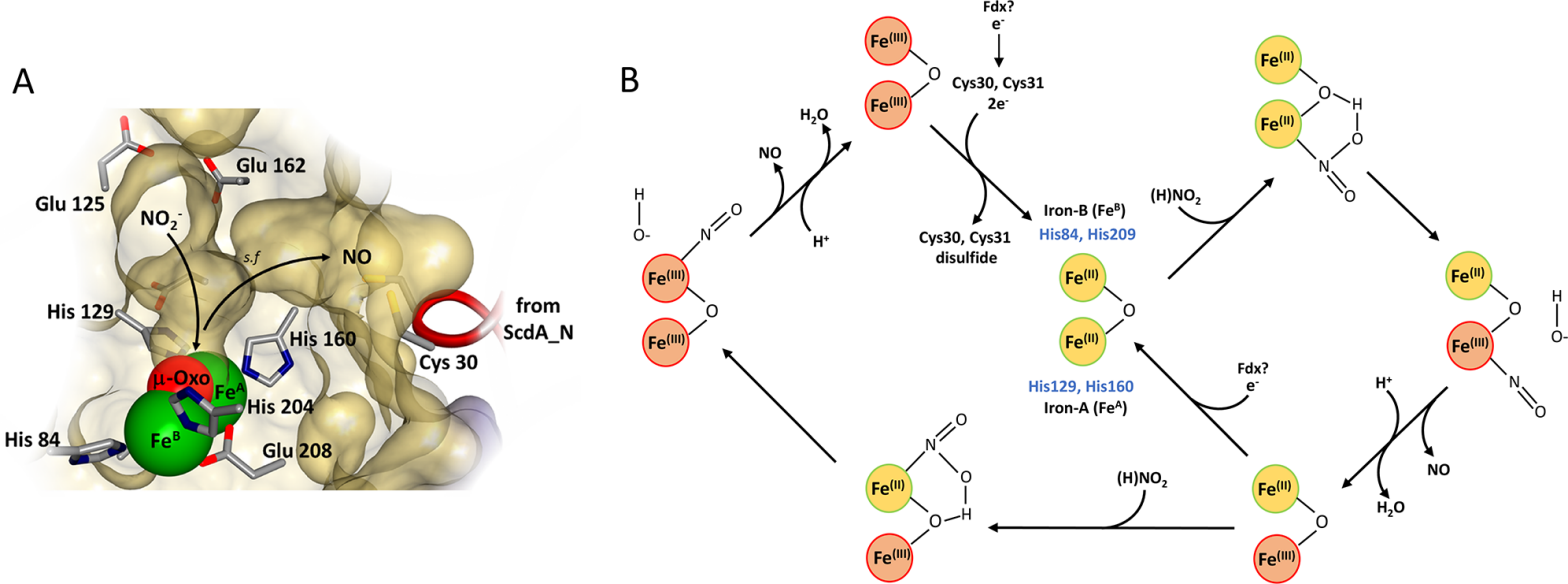
Mechanism of YtfE-catalyzed nitrite reduction. (A) The hydrophilic and hydrophobic channels connect to the di-iron cavity to create a Y-shaped cavity (1.4 Å probe radius). The surface-exposed hydrophilic channel, ringed by Glu125, Glu159, and Glu162, provides substrate access to iron A (Fe^A^) and the μ-oxo-bridge of the di-iron center. Post catalysis, the resulting NO departs via the hydrophobic channel and selection filter (*s.f.*) toward the thiolates of the ScdA_N domain. We note that the movement of the ScdA_N domain controls the access of exogenous NO to the di-iron site. Molecular graphics were analyzed and created using Biovia Discovery Studio (Dassault Systèmes). (B) Proposed mechanism of catalysis. Nitrite (or HNO_2_) binds to Fe^A^ of di-ferrous YtfE via nitrogen, promoting the oxidation of Fe^A^ and fission of HNO_2_ into NO and OH^–^. Departure of NO and OH^–^ (as H_2_O following protonation) from the mixed-valent site is followed by reduction back to the di-ferrous state or further reaction with a second (H)NO_2_ at the remaining Fe^2+^ ion. This results in a di-ferric form, which can be reduced back to the di-ferrous form by electrons from Cys30/31, resulting in a disulfide bond. Catalysis requires a supply of electrons, possibly from a ferredoxin (Fdx), which could feed into the di-iron site directly or indirectly via Cys30/31.

The crystal structure of YtfE revealed hydrophilic and hydrophobic channels that provide access to the di-iron active site cavity in a Y-shaped arrangement.^32^ The surface-exposed hydrophilic channel provides substrate access to iron A and the μ-oxo-bridge (Figure 7A). Iron B appears to be positioned outside of the active site cavity (1.4 Å probe radius). We note that the entrance of the surface-exposed hydrophilic channel is ringed with a triad of solvent-exposed glutamate residues that might act as a proton trap. The hydrophobic channel, which narrows at its confluence with the hydrophilic channel and active site cavities, potentially functions as a selection filter, providing access to the thiolates (Cys30 and Cys31) of the ScdA_N domain.^32^ Furthermore, the conformation of the ScdA_N domain relative to the hemerythrin-like domain is pivotal for controlling the access of exogenous NO to the di-iron center via the hydrophobic channel.^33^ The following is a mechanistic proposal based on the observations reported here, those previously made for nitrite reduction by hemerythrin,^46^ nonheme model complexes, and structural information on oxyanion binding to a related di-iron center.^44,63^ During catalysis, HNO_2_ or NO_2_^–^ binds to the di-ferrous form of the YtfE di-iron center in a bridging position between Fe^A^ and the μ-oxo group, as previously observed for model complexes.^63^ This promotes the oxidation of Fe^A^, resulting in production of mixed-valent YtfE bound to NO and liberation of a hydroxide anion, which upon protonation yields water.^44,46,63^ NO subsequently dissociates from the Fe^3+^ ion of the mixed-valent center and exits the protein active site, most likely via the hydrophobic channel (Figure 7B). The mixed-valent form may also be competent for nitrite reduction. The Fe^2+^ ion could bind HNO_2_/NO_2_^–^ and effect a one-electron reduction to generate NO, as described above, and di-ferric YtfE (Figure 7B).

A source of electrons is required for the di-iron center to return to its resting di-ferrous form, in which it can bind further nitrite or indeed NO. The physiological reductant is unknown, but it is likely to be a ferredoxin. Ferredoxins distribute electrons to many redox-active partners, and we note that the YtfE orthologue of *Pelobacter propionicus* contains a C-terminal auxiliary ferredoxin-like domain^39^ that contains a canonical CxxCxxCx_(*n*)_C motif typical of [4Fe-4S] ferredoxins.^52,64^

The thiolates of Cys30 and Cys31 might also function as a store of electrons for the di-iron center under certain conditions. Data for CAM-YtfE and C30A/C31A YtfE indicate that the di-Cys motif is required for catalysis under some conditions. The remaining issue is that the di-Cys motif is a two-electron reductant, while nitrite reduction at one iron is a one-electron process. The possibility that nitrite is reduced by the mixed-valent form of YtfE, resulting in di-ferric YtfE, is attractive in this regard because it would more easily account for the apparent importance of Cys30/Cys31. Their two-electron oxidation to generate a disulfide would reduce the YtfE center back to the di-ferrous form (Figure 7B), accounting for the observation that catalytic activity is dependent on Cys30/Cys31 unless electrons are available via an alternative path. Where reduction depends on Cys30/Cys31, the rate of catalysis would quickly become dependent upon the rate at which the di-Cys motif was re-reduced. Such a proposal is broadly consistent with the observed decrease in the rate of DTT-mediated reduction for C30A/C31A YtfE (Figure S3B) and the inability of this variant to catalyze iron-nitrosyl formation (Figure 6C). If the di-Cys motif is the entry point for (two) electrons, then the suggestion of ferredoxin (normally a one-electron donor) as the likely physiological reductant may seem incongruent. We note extensive literature on ferredoxin:thioredoxin reductases,^65^ which suggests that such a pathway is possible, at least mechanistically.

Nitrite reductase activity of air-exposed (mixed-valent/di-ferric) YtfE was distinct from that of the as-isolated di-ferrous form. This could be because of inefficient reduction back to the di-ferrous form and possible operation between mixed-valent and di-ferric forms. However, it is more likely that the damage/modification to the di-iron center observed upon air exposure, which included loss of the oxo-bridge or the addition of an O atom, accounts for the observed lower activity (Figure S1D). Certainly, the data give no indication that the mixed-valent/di-ferric forms are resistant to reduction back to the di-ferrous form, and so, regardless of the starting redox state of the di-iron state, we expect it would be reduced back to the di-ferrous form in the presence of a reductant.

While YtfE-generated NO is clearly able to diffuse into solution, the observation of iron-nitrosyl species (MNIC and DNIC) demonstrates that it can bind to YtfE. Such forms are not likely to be catalytically active (resulting in possible product inhibition), and so their removal might be required to maintain catalytic activity. Thus, an alternative/additional function of the di-Cys motif might be the removal of NO from the active site of YtfE. Two-electron reduction of two NO molecules at the YtfE di-iron site would result in N_2_O formation. Indeed, the capacity of YtfE to function as a NO reductase was recently demonstrated.^32,33^ We investigated the possibility that NO generated by nitrite reduction might be subsequently reduced to N_2_O but found that this happened at a very low level compared to reduction of nitrite to NO. Thus, we conclude that catalytic NO reduction is not a physiologically important role for YtfE. However, stoichiometric reduction of off-pathway YtfE iron-nitrosyl species could be.

### YtfE Is Unlikely to Function as a Source of Iron for Fe-S Cluster Repair under Physiological Conditions

Reports that YtfE can repair damaged Fe-S proteins have focused on the transfer of Fe ions from the di-iron site of YtfE to iron-deficient Fe-S proteins.^23,25^ Iron transfer/cluster reconstitution reactions were set up with 25 μM apo-ferredoxin (Fdx) in Tris buffer (pH 7.5), as previously described.^25^ We then added 50 μM YtfE, 2.5 μM IscS in the presence of 10 mM DTT, and 3 mM l-cysteine and monitored the reaction for at least 75 min. Under these conditions, no Fe-S cluster assembly was observed (Figure S7A). Extending the length of incubation by 3-fold also did not result in cluster assembly. Control reactions with ammonium ferrous sulfate as the iron source resulted in the reconstitution of an Fe-S cluster (Figure S7B).

We noted that samples of YtfE prepared for mass spectrometry in ammonium formate or triethylammonium bicarbonate (but not in sodium acetate) showed signs of damage to the di-iron site, suggesting that the di-iron site is labile in the presence of high concentrations of some low molecular weight carboxylic/carbonic acids (Figure S1B,C). Reconstitution of apo-Fdx in 250 mM ammonium formate, with YtfE as the sole iron source, was successful (Figure S7C). This suggests that holo-YtfE only acts as an iron donor for Fe-S cluster assembly under conditions that destabilize its di-iron center, and hence, it is unlikely that this is its main physiological role. The addition of 2 mM citrate to the reconstitution reaction (in Tris buffer, pH 7.5) did not support Fe-S reconstitution using holo-YtfE (Figure S7A, inset). We presume that the previous reports of YtfE acting as a source of iron for Fe-S repair^25^ were based on experiments in which the di-iron site was somehow destabilized.

### YtfE-Generated NO Can Damage Fe-S Clusters

Our experiments suggest that YtfE is unlikely to be a significant source of iron for the repair of Fe-S clusters *in vivo*, but it has also been proposed that YtfE might act as a “sponge” for endogenously produced NO, thereby lowering its concentration until it can be safely metabolized. Accordingly, the *R. eutropha* YtfE orthologue NorA has been reported to attenuate the ability of NorR transcriptional regulator to detect NO *in vivo.*(38,39,66,67) In contrast, a YtfE-dependent release of [4Fe-4S] NsrR transcriptional repression was recently demonstrated in *E. coli.*(27) Therefore, we investigated the *in vitro* effect of nitrite and NO on the [4Fe-4S] cluster of *Streptomyces coelicolor* NsrR (ScNsrR) in the presence of YtfE. ScNsrR shares ∼39% primary sequence identity with *E. coli* NsrR and, to date, is the best characterized NsrR.^8,12,45,68,69^

**Figure 8:**
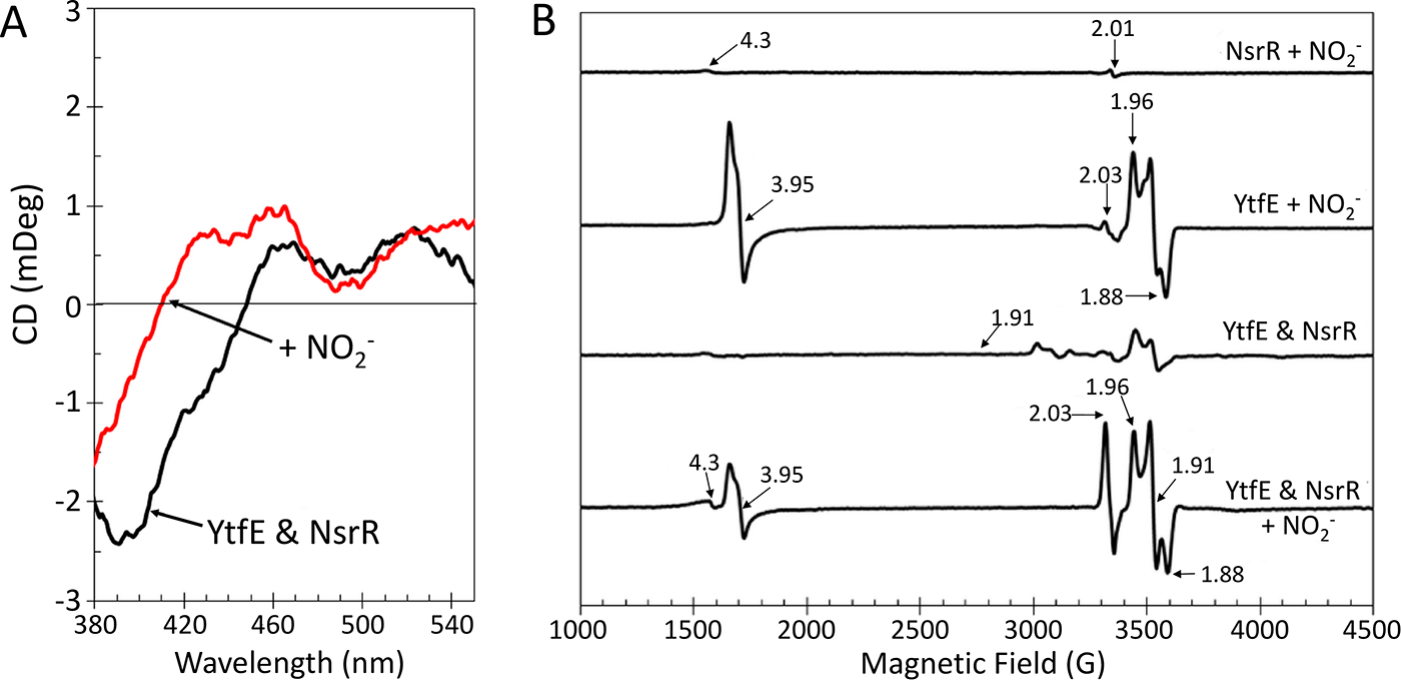
YtfE-derived NO damages Fe-S clusters. (A) CD spectrum of a deoxy YtfE (46 μM) and [4Fe-4S] NsrR (20 μM) solution before (black line) and after the addition of 3 mM KNO_2_ (red line). (B) EPR spectra of equivalent samples (100 μM deoxy YtfE and 30 μM [4Fe-4S] NsrR). NO generated by YtfE damages the [4Fe-4S] NsrR leading to DNIC formation (*g* = 2.03). In the absence of NsrR, MNIC YtfE (*g* = 3.95) was observed, along with mixed-valent YtfE. Di-ferrous YtfE in the presence of [4Fe-4S] NsrR and nitrite-treated [4Fe-4S] NsrR are shown for comparison.

The [4Fe-4S] cluster of ScNsrR is highly sensitive to the presence of NO.^8,45,68,69^ To determine whether NO produced by YtfE is able to nitrosylate ScNsrR in the solution, the visible region CD spectrum of [4Fe-4S] ScNsrR in the presence of di-ferrous YtfE was recorded and found to be identical to that recorded in its absence. The addition of excess nitrite resulted in an immediate change in the CD spectrum, where the major ScNsrR feature at (−)400 nm decreased in intensity and shifted to a shorter wavelength, as previously observed following nitrosylation of the [4Fe-4S] cluster (Figure 8A). Comparable nitrite-treated samples were also investigated by EPR spectroscopy (Figure 8B). In comparison to YtfE alone, the YtfE-NsrR mixture exhibited a larger DNIC signal (*g* = 2.03, ∼5 μM) together with lesser MNIC (*g* = 3.95, ∼10 μM) signal and a minor increase in the free hexa-aqua or adventitiously bound iron (*g* = 4.3) signal, consistent with the nitrosylation of NsrR.^45^ Similar experiments using C30A/C31A YtfE indicated that nitrosylation of some [4Fe-4S] NsrR occurred (Figure S5F), albeit to a lesser extent than observed with wild-type YtfE (Figure 8A). As a control, [4Fe-4S] NsrR (∼30 μM) was treated with nitrite; no reaction was observed, as judged by absorbance and EPR spectroscopies, even in the presence of a 1000-fold excess (30 mM) (Figure 8B and Figure S8A). The addition of nitrate also failed to modify the absorbance spectra of di-ferrous YtfE or [4Fe-4S] NsrR (not shown). To determine whether the response of [4Fe-4S] NsrR to nitrite-treated YtfE was simply a result of the production of NO catalyzed by YtfE or mediated by a protein–protein interaction, safranin O-mediated YtfE nitrite reductase activity was assayed with enough nitrite and reduced redox mediator to exceed the solubility limit (∼1.75 mM) of NO in the assay buffer within the time frame of the assay.^70^ The head space gas from the assay was then transferred to a separate cuvette containing [4Fe-4S] ScNsrR. The intensity of the (+)330 nm band in the resulting CD spectrum increased, while the band at (−)400 nm decreased in intensity, consistent with the initial reaction of ScNsrR at [NO]:[4Fe-4S] ratios of ∼2 (Figure S8B).^8,45,68,69^

To determine whether YtfE could act as a “sponge” for NO, a solution of [4Fe-4S] ScNsrR was treated with a 4-fold excess of di-ferrous YtfE. Subtraction of the contribution of di-ferrous YtfE from the resulting CD spectrum gave a difference spectrum, indicative of [4Fe-4S] ScNsrR (Figure S8C). Sequential additions up to ∼1 NO per YtfE resulted in minor changes in the ScNsrR CD spectrum (400–450 nm), whereas further additions (up to ∼3 NO per YtfE) produced changes indicative of the nitrosylation of ScNsrR (Figure S8C,D). Thus, it appears that any protection of Fe-S clusters from damage mediated by sequestration of NO by YtfE is a stoichiometric effect. Therefore, the ability of YtfE to act as a protective “sponge” *in vivo* will ultimately depend upon the concentration of YtfE present in the cell. Under aerobic conditions, *E. coli* contains a basal level of YtfE (estimated to be ∼0.6 μM YtfE, assuming 6 μM FNR).^71−73^ During anaerobic nitrate/nitrite respiration, the expression of *E. coliytfE* is enhanced.^16,40^ We note that the cytoplasmic concentration of NorA reportedly approaches ∼20 μM under denitrifying conditions, attenuating the NO-dependent activation of the NorR transcription regulator.^38,39,66,67^ Indeed, CD features of YtfE isolated from nitrite-supplemented *E. coli* cultures indicated the presence of some nitrosylated YtfE species *in vivo*. Therefore, given that cells are generally exposed to relatively low concentrations of NO, it is plausible that YtfE might also act as a cellular “sponge” for NO and might dampen the NsrR response at low NO levels. However, YtfE generates and releases NO in the presence of nitrite. Taken together, the results confirm that YtfE-generated NO is freely available in the solution to damage Fe-S clusters, consistent with recent *in vivo* observations.^27^

### Physiological Role of YtfE

YtfE and its orthologues are widely distributed among bacteria, where they have been reported to confer protection against nitrosative stress, contributing to survival within host tissues.^74,75^ In *E. coli*, the *ytfE* gene is co-regulated by NsrR with *hcp*, which encodes the hybrid cluster protein (Hcp), a high-affinity NO reductase that is essential for growth under conditions of nitrosative stress.^16,18,75^ Among enterobacteria, an *hcp* gene is readily identified in ∼90% of those that encode YtfE, consistent with a functional relationship between them (see the Supporting Information for further details). A comprehensive functional analysis of these two proteins was recently published based on studies of knockout mutants of *E. coli,* from which it was concluded that YtfE produces NO, either directly or indirectly. In the context of YtfE being important for the repair of nitrosylated Fe-S clusters, it was suggested that YtfE might somehow generate/liberate NO from nitrosylated Fe-S clusters.^27^ The *in vivo* and *in vitro* data reported here show that YtfE reduces nitrite to NO and thus are entirely consistent with the reported *in vivo* phenotypes.^27^

Nitrite, which is generated during anaerobic respiration by nitrate reductases such as NarG, may itself be toxic^3^ and also competes with nitrate for the active site of NarG, leading to its further reduction to NO.^5,76,77^ Where studied, NarG-mediated conversion of nitrite to NO appears to be more efficient than YtfE (*k*_cat_/*K*_m_ ≈ 90 × 10^4^ M^–1^ min^–1^) but with a far greater *K*_m_ (∼5 mM) for nitrite than that measured here for YtfE (∼90 μM), the latter but not the former being within the intracellular nitrite concentration range.^2^ Thus, YtfE appears to be able to compete with NarG for nitrite, particularly when the preferred substrate for NarG, nitrate, is plentiful. Under such conditions, e.g., when the nitrate/nitrite ratio is high, the cytoplasmic siroheme-containing nitrite reductase (NirB) is produced. NirB is highly active (*K*_m_ ≈ 6 μM, *k*_cat_/*K*_m_ ≈ 183 × 10^6^ M^–1^ s^–1^) in reducing nitrite to ammonia.^78,79^ Thus, NirB is a much more efficient nitrite reductase than YtfE, but YtfE might still compete for nitrite because NirB is only produced under high concentrations of nitrate/nitrite.^80^

**Figure 9:**
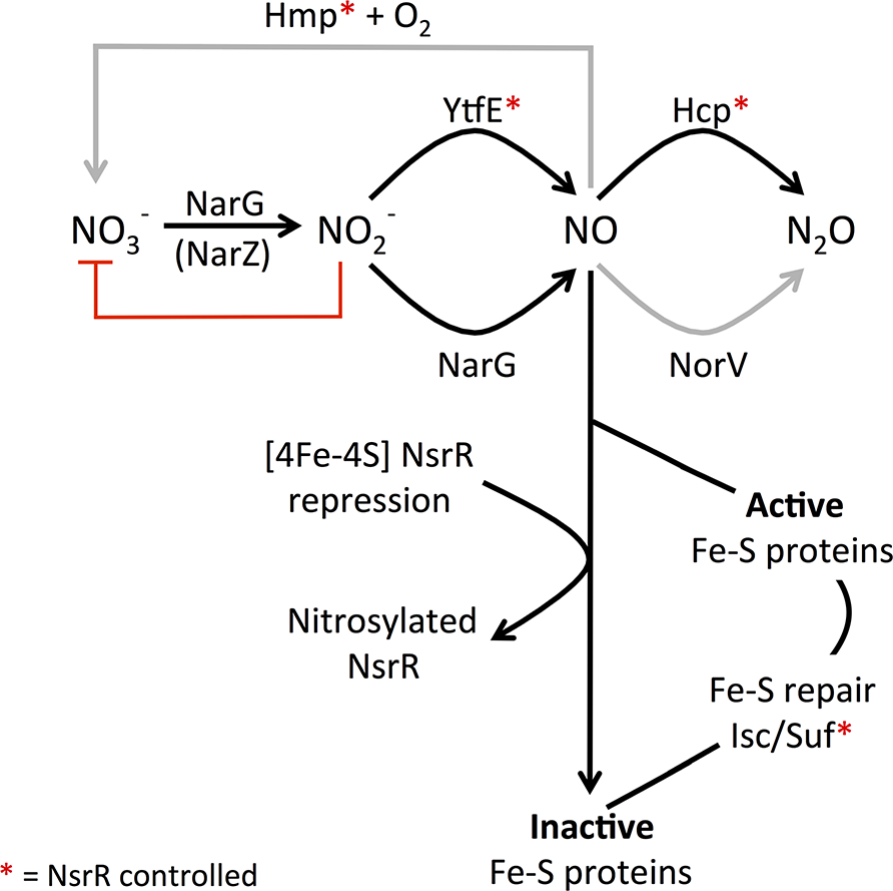
Proposed role of YtfE in the management of endogenous nitrosative stress in *E. coli*. Nitrate reductases (NarG and, probably, NarZ) increase the cytoplasmic concentration of nitrite. Accumulated nitrite competes with nitrate at the active site of NarG and therefore impairs nitrate reduction and growth (red line). We propose that YtfE reduces nitrite to NO to optimize nitrate respiratory growth. The NO generated by YtfE can inactivate Fe-S proteins, including the global transcriptional repressor [4Fe-4S] NsrR. Nitrosylated NsrR is not a competent repressor, leading to the derepression of genes encoding the response to nitrosative stress (indicated by red asterisks), including the high-affinity NO reductase Hcp and its cognate reductase Hcr. Thus, YtfE and NsrR can be thought of as a two-component system that monitors cytosolic nitrite concentrations, resulting in responses, including the induction of *hcp-hcr*, that promote efficient nitrate respiratory growth, with Hcp acting predominantly under anaerobic conditions when the concentration of NO is <1 μM. The alternative NO reductases, Hmp and NorV, lower the cytosolic concentration of NO under different regimes, microaerobic (Hmp) or anaerobic with >1 μM NO (NorV), respectively, (gray arrows). Under some conditions, NirB is deployed to convert nitrite to ammonium (see the main text). Inactivated Fe-S proteins are repaired by the Isc and/or Suf systems.

We propose that, in *E. coli*, excess intracellular nitrite, as well as being exported and reduced to ammonia by NirB, is reduced to NO by YtfE. The resulting NO is sensed directly by [4Fe-4S] NsrR, which results in upregulation of the *hcp-hcr* operon encoding the high-affinity NO reductase Hcp and its partner reductase, Hcr.^18^ Thus, the combination of YtfE and Hcp-Hcr functions to detoxify cytoplasmic nitrite, which, if left unchecked, would accumulate and inhibit nitrate reduction and, consequently, respiration and growth (Figure 9). Hcp is also believed, somewhat controversially,^28,29^ to be involved in the S-thiol nitrosation of proteins when cellular NAD^+^/NADH ratios are high.^20,81^ Such a process requires a source of NO, which was suggested to be supplied by Nar acting on nitrite. This may be correct under some conditions, but anaerobic cultures of *E. coli* provided with isotopically labeled nitrate and nitrite (2:1 ratio) produced N_2_O from nitrite, before nitrate reduction began, suggesting that NO was produced independently of Nar.^82^ While some aspects remain controversial, these reports are consistent with the proposal that YtfE in partnership with Hcp-Hcr plays a key role in limiting the accumulation of both nitrite and NO in the bacterial cytoplasm while possibly facilitating protective S-nitrosation when the nitrate/nitrite ratio is high.

The connection between YtfE and the repair of Fe-S cluster proteins is most likely indirect; through the alleviation of NsrR-mediated repression of the Suf Fe-S cluster biogenesis system (*sufABCDSE*), which is the principal route for production of Fe-S clusters under stress conditions, as well as genes encoding proteins that function to counter nitrosative stress (Figure 9).^14,83^ We also note that the catalytic activity of aconitase and fumarase are reversibly/competitively inhibited by nitrite.^84^

### Concluding Remarks

We have demonstrated that YtfE is a nitrite reductase that reduces nitrite to NO under conditions that are physiologically relevant. Based upon the coordinate regulation of *ytfE* and *hcp-hcr* expression by the NO-sensitive repressor NsrR, we propose that YtfE catalyzes the first reaction of a two-step pathway that prevents the accumulation of both nitrite and NO in the bacterial cytoplasm under conditions typically encountered in the animal gastrointestinal tract. An obvious question that arises concerns the generation of a toxic product (NO) as the first step of the detoxification process. Similar issues relate to the production of NO during bacterial denitrification,^85^ and we suggest that the high-affinity two-step pathway provided by YtfE in partnership with Hcp-Hcr prevents the accumulation of NO at levels that might damage cell components.

Finally, we note several reports that many of the proteins mentioned above interact to form supramolecular complexes or so-called interactomes.^20,81^ YtfE was identified as part of the nitrate reductase interactome, leading to the suggestion that the localization of proteins involved in nitrate respiration and NO homeostasis might promote maximal electron flux and minimal toxicity.^81^ The data reported here, together with previously reported *in vitro* and *in vivo* studies of *ytfE*/YtfE, lead us to propose that the YtfE-catalyzed reduction of nitrite (or HNO_2_) to NO is of physiological significance.

## Methods

### NO-Induced Transcription in Response to Nitrite and YtfE

For *in vivo* transcription studies, strains RK4353 (*ytfE^+^*) and JCB5211 *(*Δ*ytfE*) (see Table 2) were transformed with the NsrR reporter plasmid pNF383.^27^ Briefly, pNF383 features β-galactosidase under the control of the *hcp* promoter region, thus making it dependent upon relief of NsrR repression by cytoplasmic NO.^6^ Duplicate anaerobic cultures (100 mL) were grown at 37 °C in minimal salts medium, supplemented with glycerol and fumarate, as previously described.^27^ When OD_650 nm_ reached 0.2, one culture was supplemented with 2.5 mM NaNO_2_, while the other culture served as a control. Cultures were incubated without shaking for an additional 2 h. Samples were removed as indicated, and β-galactosidase activities were measured according to the Miller protocol.^86^ Similar results were obtained in biological replicate experiments, with independent cultures grown on different days. Data were analyzed for statistical significance using unpaired samples *t*-test using Prism (GraphPad Software, v5). Results were considered statistically significant where *p* ≤ 0.05.

**Table 2:** Strains and Plasmids

	description	reference or source
strain		
RK4353	parent strain: *lacU169 araD139 rpsL gyrA non*	(87)
JCB5211	RK4353 Δ*ytfE*::cat	(24)
JCB5270	RK4353 Δ*narGHJI*, *narZ*, *hcp*, *norVW*, *nrfAB*, *nirBD*, and *hmp*	(27)
JCB5280	Δ*ytfE*::cat derivative of JCB5270	(27)
plasmid		
pNF383	*hcp* regulatory region ligated into pRW50 to give a P*hcp*::*lacZ* transcriptional fusion; Tet^*R*^	(6, 16)
pBB2016	a low copy pACYC184 derivative expressing *ytfE* under the control of its own promoter; Tet^*R*^ and Cm^*R*^	(18, 88)
pGS2618	a pET24a derivative for overexpression of His-tagged *ytfE*; Kan^*R*^	(27)
pGS2618CA	a derivative of pGS2618 for overexpression of the Cya30Ala/Cys31Ala YtfE variant; Kan^*R*^	this work, Genscript

### Effect of YtfE on Growth in the Presence of Nitrite

To monitor the effect of YtfE on growth, anaerobic cultures of JCB5270 (*ytfE^+^*) or JCB5280 (Δ*ytfE*) (see Table 2) were grown in minimal salts medium, as previously described.^27^ When OD_650 nm_ reached 0.2–0.4, the cultures were supplemented with 1 mM NaNO_2_ and OD_650 nm_ was tracked for ∼6 h. To confirm that growth inhibition was YtfE-dependent, JCB5270 and JCB5280 were transformed with a low copy number plasmid, pBB2016, which expresses *ytfE* under the control of its own promoter. Unsupplemented cultures served as controls. Similar results were obtained in biological replicate experiments, with independent cultures grown on different days. Data were analyzed for statistical significance as above, and results were considered statistically significant where *p* ≤ 0.05.

### Protein Expression and Anaerobic Purification

C-terminally His-tagged *E. coli* YtfE was expressed from pGS2618 in *E. coli* BL21 (DE3)-T1^R^.^27^ Cultures were grown aerobically in 2.5 L of Miller’s LB broth, supplemented with kanamycin (50 mg L^–1^) at 37 °C. When the OD_600 nm_ reached 1.2, protein production was induced by the addition of 50 μM isopropyl β-d-1-thiogalactopyranoside (IPTG). Cultures were supplemented with 20 mM NaNO_3_ or 5 mM KNO_2_, 200 μM ferric ammonium citrate, 25 μM l-cysteine, and 25 μM l-methionine and grown overnight at 23 °C, with 65 rpm shaking. Bacteria were harvested by centrifugation, washed with lysis buffer (50 mM Tris, 100 mM NaCl, and 5% (v/v) glycerol, at pH 8.0), and stored at −80 °C until needed. Unless otherwise stated, all subsequent purification steps were performed in an anaerobic cabinet with [O_2_] < 5 ppm (Belle Technology).

Cell pellets were suspended in lysis buffer (75 mL) with the addition of lysozyme (0.4 mg mL^–1^), DNaseI (0.08 mg mL^–1^), 2 mM phenylmethylsulfonyl fluoride (PMSF), and 1.3% (v/v) ethanol. The cell suspension was removed from the anaerobic cabinet, sonicated twice while on ice, and returned to the anaerobic cabinet. The cell lysate was transferred to O-ring sealed centrifuge tubes (Nalgene) and centrifuged outside of the cabinet at 40,000*g* for 45 min at 1 °C. The supernatant was immediately loaded onto a HiTrap Ni^2+^-chelating column (2 × 5 mL; Cytiva) previously equilibrated with lysis buffer. The column was washed with 50 mM Tris, 1 M NaCl, 5% (v/v) glycerol (pH 8.0), and then lysis buffer. Bound proteins were eluted using a linear gradient from 0 to 100% (v/v) lysis buffer containing 200 mM l-histidine. Fractions containing YtfE (identified by SDS-PAGE) were pooled, diluted 5-fold with 50 mM Tris, 10 mM NaCl, and 5% (v/v) glycerol (pH 8.0), loaded onto a HiTrap Q column, washed with lysis buffer, and eluted using lysis buffer containing 2 M NaCl. Fractions containing YtfE were pooled and stored in an anaerobic freezer until needed. A Cys30Ala/Cys31Ala YtfE variant (pGS2618CA, Genscript) was expressed and purified in the same way.

Carboxymethylated YtfE was prepared in assay buffer (100 mM Tris, pH 8.0) by reducing YtfE with a 2-fold excess of tris(2-carboxyethyl)phosphine (TCEP) and subsequently adding a 30-fold excess of iodoacetamide. Unreacted iodoacetamide was removed with a PD10 desalting column. LC–MS was used to confirm successful alkylation. His-tagged *S. coelicolor* NsrR, holo- or apo-, was purified as previously described.^45^

### Reduction of YtfE by Dithiothreitol

An aliquot (30 μL) of YtfE was exposed to air for 15 min, returned to the anaerobic cabinet, and diluted to 50 μM with assay buffer (see above), and then, the absorbance spectrum was recorded. Dithiothreitol (DTT, 10 mM final concentration) was added and the solution was incubated at room temperature for 75 min. After incubation, excess DTT and other small molecules (≤5 kDa) were removed from the now colorless YtfE by a PD10 column. Early eluting fractions, containing YtfE, were re-exposed to air and quantified using ε_280 nm_ = 24.26 mM^–1^ cm^–1^ and ε_340 nm_ = 4.00 mM^–1^ cm^–1^ for the protein and di-iron site, respectively.^25^ Late eluting fractions were assayed for the presence of iron with Ferene, as previously described.^89^ To investigate the kinetics of DTT-mediated reduction, the disappearance of the *A*_340 nm_ band present in the spectrum of air-exposed YtfE (∼90 μM) was monitored at increasing concentrations of DTT (0 to 80 mM). Initial rates, expressed as Δ*A*_340 nm_ min^–1^, were determined and plotted as a function of the DTT concentration. The experimental data could be fitted using a simple binding isotherm, from which a *K*_d_ was obtained. Reactions in which DTT was replaced with physiological concentrations of glutathione (3 mM) or NADH (0.25 mM) were conducted for comparison.^90^

### Spontaneous Reduction of Nitrite by Di-Ferrous YtfE

Potassium nitrite (3 mM final concentration) was added to ∼100 μM di-ferrous YtfE in anaerobic assay buffer (see above). Excess nitrite and other small molecules (≤5000 Da) were removed by a PD10 column and the absorbance spectrum was recorded. Difference spectra, generated by subtracting the spectrum of air-exposed YtfE, were obtained. Nitrite-treated di-ferrous YtfE was analyzed by EPR and native mass spectrometry. Control reactions containing DTT, nitrite, and ferrous ammonium sulfate in place of YtfE were also analyzed. The spontaneous and rapid formation of iron-nitrosyl complexes (λ_max_ = 360 nm) in solutions containing NO, a suitable ligand, and “free” iron is well documented,^55−58^ and it was used as a reporter for the production of NO. Thus, to investigate the kinetics of YtfE-mediated NO production, the increase in absorbance at 360 nm was used to detect *in situ* iron-nitrosyl formation and track the reaction kinetics when YtfE (10 μM) was exposed to increasing concentrations of KNO_2_ (0 to 1.5 mM) in the presence of excess glutathione (3 mM) and Fe^2+^ (200 μM). Initial rates, expressed as Δ*A*_360 nm_ min^–1^, were determined and plotted as a function of KNO_2_ concentration. The experimental data could be fitted using a simple binding isotherm from which *K*_m_ and Δ*A*^max^ were obtained. *V*_max_ (μmol NO min^–1^ mg^–1^) was determined using the extinction coefficient of 7.4 mM^–1^ cm^–1^ for Roussin’s red ester of glutathione (RRE, [Fe_2_(NO)_4_(GSH)_2_]).^59^ Using the molecular mass of YtfE (26,154 Da), *k*_cat_ (min^–1^) could be approximated. The catalytic efficiency, *K*_m_/*k*_cat_, of nitrite reduction by YtfE was also determined. Control reactions lacking di-ferrous YtfE failed to generate a colored species over the same time frame.

### Nitrite Reductase Assay

The nitrite reductase activity of YtfE was measured spectrophotometrically via the nitrite-dependent oxidation of reduced methyl viologen (ε_600 nm_ = 13.70 mM^–1^ cm^–1^).^53^ Briefly, an anaerobic cuvette containing dithionite-reduced methyl viologen (58 μM) and di-ferrous YtfE (as-isolated, 10 μM) in assay buffer was injected with an aliquot (5 μL) of KNO_2_ (0 to 1.5 mM, final concentration) and *A*_600 nm_ was monitored for up to 100 s. To determine the pH dependence of the reaction, methyl viologen assays were conducted at pH 7.0, 7.5, and 8.0. To determine the effect of the redox mediator potential, assays were repeated at pH 7.5 using safranin O (ε_518 nm_ = 37.05 ± 2.1 mM^–1^ cm^–1^). To probe the specificity for NO_2_^–^, assays were repeated in the presence of 1.5 mM KNO_3_. Control nitrite reduction assays containing 1.5 mM KNO_2_ but lacking YtfE failed to oxidize the redox mediator over the same time frame.

Initial rates, expressed as Δ*A*_600 nm_ min^–1^, were determined using the kinetics module of Spectra Analysis (version 1.53.04, Jasco) and plotted as a function of the KNO_2_ concentration. The experimental data could be fitted to simple Michaelis–Menten kinetics using the following equation, *y* = (Δ*A*_max_ × [S])/(*K*_m_ + [S]), from which *K*_m_ and Δ*A*_max_ (maximum rate) were obtained. *V*_max_ (μmol min^–1^ mg^–1^) was determined using the extinction coefficient for methyl viologen or safranin O. *k*_cat_ (and *K*_m_/*k*_cat_) was determined as above. A minimum of two biological repeats and two technical repeats were averaged.

### Detection of N_2_O as a Product of Nitrite Reduction

A solution of di-ferrous YtfE (0.5 mL, ∼437 μM) was prepared using anaerobic assay buffer (100 mM Tris, pH 7.5) in 3 mL screw cap Exetainer vials (Labco) that had been opened to the glovebox atmosphere. The reaction was initiated by injecting an aliquot of KNO_2_ (5 μL) with a 6.14 mM final concentration. The reaction was allowed to proceed for 15 min before the headspace of the vial was removed (2.5 mL was removed and replaced with 2.5 mL N_2_) and stored in a 3 mL pre-evacuated Exetainer vial. In some cases, reactions contained 2.7 or 5.4 mM DTT as a reductant. Reactions carried out in the absence of YtfE or with oxidized YtfE (mixed-valent and/or di-ferric) served as controls. The reaction vials were incubated for an additional 4 h before the headspace was sampled again. Measurements of N_2_O levels were made by gas chromatography of 50 μL headspace samples injected (Samplelock syringe, Hamilton) onto an Elite-PLOT Q capillary column (30 m × 0.53 mm i.d., 20 m film thickness) fitted to a Clarus 500 gas chromatographer (PerkinElmer) with an electron capture detector. The carrier was N_2_, with make-up gas of 95 and 5% (v/v) argon and methane, respectively). The instrument was calibrated using standards of N_2_O containing 5, 100, 1000, 5000, and 10,000 ppm N_2_O in N_2_ (Air Liquide UK). Total N_2_O amounts in liquid and gaseous phases were calculated by applying Henry’s Law constant for N_2_O at 30 °C and a *K*_H_^cc^ of 0.5392.^91^

### YtfE-Mediated Fe-S Cluster Repair

*E. coli* IscS and ferredoxin (Fdx) were overexpressed and purified as previously described.^92−95^ Briefly, His-tagged glutathione S-transferase fusions of IscS or Fdx were isolated by Ni^2+^-affinity chromatography and tag-free IscS and Fdx released with tobacco etch virus protease (TEV), confirmed by LC–MS, and quantified by absorbance spectroscopy using ε_280 nm_ = 41.37 or 6.99 mM^–1^ cm^–1^ for IscS and Fdx, respectively.^92−95^ Apo-Fdx was prepared as previously described^25^ or by treating Fdx with 5 mM EDTA for 2 h, followed by desalting to remove low molecular weight species.^95,96^ ICP-MS (iCAP-TQ, Thermo Fisher Scientific) was used to confirm the removal of Fe ions from apo-Fdx. The purity of the sample was confirmed using LC–MS. Reconstitution reactions, containing apo-Fdx (25 μM) with l-cysteine (3 mM), dithiothreitol (10 mM), and 50 μM di-ferrous YtfE (equivalent to ∼100 μM Fe), were prepared in anaerobic buffer (20 mM Tris and 150 mM NaCl, pH 7.5) and initiated by the addition of *E. coli* IscS (2.5 μM).^25^ Absorbance (*A*_420 nm_) due to newly synthesized Fe-S clusters was used to track the progress of the reactions at 15, 30, 75, 90, and 200 min. Control reactions, lacking di-ferrous YtfE, were supplemented with 100 μM ferrous ammonium sulfate.

### The Effect of YtfE on [4Fe-4S] NsrR

To determine the extent of “free” NO generated by YtfE from nitrite, a solution (2 mL) containing [4Fe-4S] NsrR (20 μM) and di-ferrous YtfE (46 μM) in assay buffer (see above) was analyzed by CD spectroscopy. An aliquot of KNO_2_ was injected to give a final concentration of 3.65 mM and the spectrum was remeasured. Comparable samples (33 μM [4Fe-4S] NsrR and 100 μM di-ferrous YtfE) were also analyzed by EPR spectroscopy and compared to YtfE titrated with increasing amounts of the NO-releasing reagent Proli-NONOate. As a control, [4Fe-4S] NsrR (∼30 μM) was treated with increasing amounts of KNO_2_. To determine whether YtfE confers protection against free NO, a solution (2 mL) containing [4Fe-4S] NsrR (64 μM) and di-ferrous YtfE (300 μM) in assay buffer was analyzed by difference CD spectroscopy. The contribution from di-ferrous YtfE was subtracted to reveal the CD spectrum of [4Fe-4S] NsrR. The resulting solution was then titrated with increasing amounts of the NO-releasing reagent Proli-NONOate, and the response of the (−)450 nm band (indicative of [4Fe-4S] NsrR) was monitored. The previously published changes in the CD spectrum of [4Fe-4S] NsrR in response to NO were used as a guide to spectral changes in the presence of excess YtfE.^45,69^

In a separate reaction, the headspace of a safranin O-mediated nitrite reduction assay was transferred to a separate buffered solution of [4Fe-4S] NsrR. Briefly, a solution of di-ferrous YtfE (1 mL, 46 μM) was treated with 6 mM reduced safranin O and 6 mM KNO_2_. The reaction was allowed to proceed for 100 s before the headspace was removed by a syringe (3 mL was removed and replaced with 3 mL N_2_) and bubbled through a solution (2 mL) of [4Fe-4S] NsrR (58 μM), which was incubated for 10 min at 37 °C prior to measurement.

### Mass Spectrometry

The mass of YtfE was determined by routine liquid chromatography–mass spectrometry (LC–MS) analysis.^97^ Briefly, YtfE was diluted to ∼10 μM with an aqueous mixture of 2% (v/v) acetonitrile and 0.1% (v/v) formic acid in an LC–MS vial, and the sample loaded onto a Proswift RP-1S column (4.6 × 50 mm) (Thermo Scientific) associated with an Ultimate 3000 uHPLC system (Dionex, Leeds, UK). Bound proteins were eluted (0.2 mL min^–1^) using a linear gradient (15 min) from 2 to 100% (v/v) acetonitrile and 0.1% (v/v) formic acid. The eluent was continuously infused into a Bruker microQTOF-QIII mass spectrometer, running Hystar (Bruker Daltonics, Coventry, UK), using positive mode electrospray ionization (ESI).

For native mass spectrometry, YtfE samples were buffer-exchanged into either 200 mM ammonium acetate (pH 8.0), 200 mM ammonium formate (pH 8.0), or 50 mM triethylammonium bicarbonate (pH 8.5) using a PD minitrap G25 or PD10 desalting column (Cytiva). Samples were diluted to ∼25 μM and infused directly (0.3 mL h^–1^) into the source of the mass spectrometer operating in positive mode. The spectra (1800–3200 *m*/*z*) were recorded for 5 min with acquisition via Bruker oTOF control software, with the following parameters: dry gas flow, 4 L min^–1^; nebulizer gas pressure, 0.8 bar; dry gas temperature, 180 °C; capillary voltage, 3500 V; offset, 500 V; quadrupole ion voltage, 5 V; collision RF, 650 Vpp; collision cell voltage, 10 V. Note that the overall charge of the metal-containing cofactor must be considered to properly assign a molecular mass from the *m*/*z* spectrum. For iron-containing proteins, the peaks correspond to [*M* + [Fe]^*x*^ + (*z* – *x*)*H*]/*z*, where *M* is the molecular mass of the protein, [Fe] is the mass of the metallo-cofactor with *x* charge, *H* is the mass of the proton, and *z* is the total charge of the ion. In this expression, the charge of the metallo-cofactor, *x*, offsets the number of protons required to obtain an ion with *z* charge.^97−100^ Processing and analysis of MS data were carried out using Compass Data Analysis version 4.1 (Bruker Daltonik, Bremen, Germany). The neutral mass spectra were generated using the ESI Compass version 1.3 Maximum Entropy deconvolution algorithm. Exact masses are reported from peak centroids, representing the isotope average neutral mass.

### Spectroscopy and Other Procedures

UV–visible absorbance and kinetic measurements were made using a Jasco V550 spectrophotometer. The circular dichroism (CD) spectra were measured with a Jasco J810 spectropolarimeter. The EPR spectra were recorded on a Bruker Elexsys-II E580 fitted with a SHQ resonator and an ER 4112 HV low temperature control system operating at 10 K. Electron paramagnetic resonance (EPR) samples (250 μL) were frozen in liquid nitrogen and run with the following parameters: microwave frequency, 9.42–9.44 GHz; microwave power, 0.2 mW; modulation amplitude, 0.5 mT; same receiver gain. Quantification of spin concentrations in paramagnetic samples was achieved by double integration of EPR signals and comparison with the signals of spin concentration standards: 100 μM Cu^2+^ in 10-fold excess EDTA, 100 μM Fe^3+^-EDTA, prepared as previously described,^101^ or 100 μM Fe^2+^-NO EDTA, prepared by addition of excess nitrite and ascorbate to a Fe^2+^ solution, which yielded an EPR spectrum containing only the *S* = 3/2 MNIC signal. In the latter case, it was assumed that zero-field splitting was similar in the sample and standard.^102,103^ Where appropriate, the EPR spectra were simulated with Easyspin 5.2.33 in Matlab.^104^

The total protein concentration was determined by the method of Pierce with bovine serum albumin as the standard. The purity of the protein was checked using SDS-PAGE and LC–MS. The iron content was determined colorimetrically with Ferene (Sigma-Aldrich) by reference to a calibration curve generated from Fe^3+^ solutions prepared from a SpectrosoL standard iron solution.^105^ iCAP-triple quadrupole-inductively coupled plasma-mass spectrometry (iCAP-TQ-ICP-MS; Thermo Fisher Scientific) was used to assay for S, Fe, Zn, Mn, Cu, Ni, and Mo.^106,107^ As-isolated YtfE was found to be ≥98% replete with iron.

## Supporting Information Available

Characterization and redox cycling of YtfE; N_2_O and NO production by YtfE; bioinformatic analyses; additional mass spectrometric, spectroscopic, and kinetic results (PDF)

## Notes

**Data Availability.** All relevant data are presented in the main paper or in Supporting Information, and are available from the authors upon reasonable request.
