# Supplementary material for: The Di-Iron Protein YtfE Is a Nitric Oxide-Generating Nitrite Reductase Involved in the Management of Nitrosative Stress
Source: J Am Chem Soc. 2022 Apr 13;144(16):7129–45. doi: 10.1021/jacs.1c12407 (PMC9052748; doi:10.1021/jacs.1c12407)
Supplement: Supplementary file 1 — ja1c12407_si_001.pdf [file ja1c12407_si_001.pdf]

Supporting Information

The di-iron protein YtfE is a nitric oxide-generating nitrite reductase involved in management of nitrosative stress

Jason C. Crack,[†,*] Basema K. Balasiny,[‡] Sophie P. Bennett,[†] Matthew D. Rolfe,[§] Afonso Froes,[†] Fraser MacMillan,[†] Jeffrey Green,[§] Jeffrey A. Cole,[‡] and Nick E. Le Brun[†,*]

[†]Centre for Molecular and Structural Biochemistry, School of Chemistry, University of East Anglia, Norwich Research Park, Norwich, NR4 7TJ, UK.

[‡]Institute of Microbiology and Infection and School of Biosciences, University of Birmingham, Birmingham B15 2TT, UK;

[§]School of Biosciences, University of Sheffield, Sheffield, S10 2TN, UK.

* Corresponding authors J.crack@uea.ac.uk; N.Le-brun@uea.ac.uk

SUPPORTING RESULTS

Characterization of anaerobically purified YtfE. Anaerobically prepared YtfE from nitrate-supplemented cultures was colorless (pale pink when concentrated) with weak, poorly resolved absorption bands at ≥300 nm, consistent with the presence of di-ferrous, Fe^{2+}/Fe^{2+} (deoxy) YtfE[1-2] (Fig. 3A). Circular dichroism spectroscopy is more sensitive to the optical transitions of metallo-proteins than absorbance spectroscopy, and is particularly sensitive to changes in the local environment or redox state of metallo-cofactors.[3-5] The CD spectrum of as isolated YtfE was broad with two positive features at (+)345 nm and (+)440 nm, and a single negative feature at (-)560 nm (Fig. 3B).

The di-iron site of YtfE, as observed for hemerythrin, is also capable of ligating other metal ions, including Zn and Mn.[6] Elemental analysis by ICP-MS showed that YtfE contained an average of 2.09 (± 0.19) Fe, 0.21 (± 0.02) Zn, and 0.17 (± 0.01) Mn atoms per protein. The LC-MS spectrum of purified YtfE, in the absence of reductant, contained a major peak at 26,030 Da, corresponding to the metal-free (apo) protein with the N-terminal methionine residue removed (predicted mass 26,030 Da, see Fig. 3C, Table S1).

Non-denaturing ESI-MS (native MS), in which non-covalently bound cofactors are retained upon ionization, has been shown to be extremely useful for determining the nature of metallo-cofactors and protein complexes.[7-11] Application here gave a m/z spectrum dominated by monomeric YtfE, with trace amounts of dimeric (~11% relative abundance) together with some higher order species (Fig. S1A), consistent with solution studies.[6, 12] The deconvoluted neutral mass spectrum for monomeric YtfE (as isolated from nitrate-supplemented cells) contained two significant peaks. The first, at 26,154 Da, corresponded to YtfE with a μ-oxo bridged di-iron cofactor (Fig. 3C; predicted mass 26,156 Da, Table S1). The absorbance properties and featureless EPR spectrum of as isolated YtfE demonstrate that it is in the di-ferrous form (Fig. 3).

The second peak represented an acetate/formate or carbonate adduct that depended on the buffer used (Fig. 3C, Fig. S1B, C and Table S1). In ammonium acetate, a peak at 26,213 Da, corresponding to YtfE plus an acetate adduct (+59 Da), was observed (Fig. 3C); in ammonium formate buffer, the peak was at +44 Da, due to a formate adduct (Fig. S1B); and, in tetramethylammonium bicarbonate, the peak was at +61 Da due to a carbonate adduct (Fig. S1C). In the latter case, the YtfE peak was shifted by -3 Da to 26,151 Da, indicative of oxidation of the di-iron site along with the presence of an intra-molecular disulfide resulting from the oxidation of the two Cys residues of the N-terminal domain (Table S1). Two lower intensity peaks were also observed at 26,160 and 26,207 Da, corresponding to the full length protein (+131 Da relative to the major peak, due to incomplete N-terminal methionine residue removal) and N-terminal gluconylation (+178 Da), respectively.[13-14]

Redox cycling of the YtfE di-iron site. Limited exposure of as isolated di-ferrous YtfE to air resulted in significant darkening of the sample, with an associated absorption feature at 340 nm, together with weaker absorption features at 500–520 nm (giving an orange/red color when concentrated)[2] (Fig. 3A). The absorption characteristics of the 340 nm feature, $\varepsilon_{340\ nm}$ = 4.00 (±0.5) mM^{-1} cm^{-1}, were similar to those previously assigned to the mixed valent (Fe^{3+}/Fe^{2+}) or di-ferric (Fe^{3+}/Fe^{3+}) states of YtfE and other hemerythrin-like proteins [3, 15]. Similar spectra were obtained using potassium ferricyanide (6-fold excess) or ammonium persulfate (10-fold excess) as oxidant. We note that the ~500 nm feature in hemerythrin likely originates from an oxy form (formally Fe^{3+}/Fe^{3+}-OOH, 2.3 mM^{-1} cm^{-1}) or the di-ferric state (0.2 mM^{-1} cm^{-1}).[3, 6, 15] As isolated, YtfE gave a featureless EPR spectrum (Fig. 3D). Exposure to air resulted in the observation of signals at g = 1.96, 1.91, and 1.88 that are characteristic of a S = ½ mixed valent, Fe^{3+}/Fe^{2+} di-iron center, as previously reported (Fig. 3D, Fig. S2A, Table S2).[2] Spin quantification revealed that this accounted for 37% of the YtfE protein concentration, with the remainder EPR silent. Hence, ~60% of the sample is present as di-ferric YtfE following brief (15 min) exposure to air, with the remaining ~40% in the mixed valent form. Significant changes in the CD spectrum were also observed upon exposure of di-ferrous YtfE to ambient O_2 for 15 min, with weak features appearing at (+)320, 430, and 480 nm, and at (-)370 and 400 nm (Fig. 3B, inset, red trace).

Using non-denaturing mass spectrometry, exposure of di-ferrous YtfE in ammonium acetate to ambient O_2 gave poorly resolved major peaks between 26,150 and 26,153 Da, likely corresponding to mixed valent and di-ferric YtfE along with a disulfide (full oxidation of the di-iron site and Cys30,31 would result in a -4 Da shift, see Fig. S1D and Table S1).

Anaerobic addition of dithionite to air-exposed, oxidized YtfE resulted in a CD spectrum similar to that of the as isolated protein (Fig. 3B, inset, green trace), consistent with initial isolation of di-ferrous YtfE and

the ability of the cofactor to undergo redox cycling. Previous studies of YtfE as an iron source for cluster reconstitution employed DTT as reductant. Addition of DTT (10 mM) to air-exposed YtfE (~100 μM di-iron center) resulted in YtfE becoming colorless. Removal of low molecular weight species (<5 kDa) and re-exposure to air resulted in the reappearance of the 340 nm and 520 nm features in the absorbance spectrum, consistent with facile redox-cycling of the di-iron center, with no significant loss of iron detected (Fig. S3A). The dependence of the rate of reduction on DTT concentration was consistent with a relatively weak interaction between DTT and YtfE, see Fig. S4. The rates of reduction by 10 mM ascorbate or 0.2 mM NADH were only 20% or 10%, respectively, of that observed for DTT. Reduced glutathione (3.5 mM) was completely ineffective in reducing oxidized YtfE (Fig. S3B, C), suggesting that accessibility of the reductant to the protein/di-iron center (and not just reduction potential) is important for reduction to occur.

Direct comparison of N_2O and NO production by YtfE. Nitrite (6 mM) was added to a solution of di-ferrous YtfE (437 μM) in the absence of a reductant and incubated for 15 min. Headspace gas and solution were analyzed for N_2O by GC and for NO (in the form of MNIC species) by EPR spin quantification. N_2O was at the lower detection limit, corresponding to 0.007 mol per mol of YtfE, while $S = {}^3/_2$ MNIC spin concentration corresponded to ~0.3 mol per mol of YtfE. This represents a lower limit of NO generated, because NO does not bind very tightly to the YtfE di-iron center and readily diffuses into solution. Indeed, a titration of di-ferrous YtfE with NO indicated that the MNIC signal did not saturate until addition of ~8 NO per YtfE (Fig. S2). YtfE-promoted formation of NO by reduction of nitrite results in oxidation of the di-iron center, to mixed valent and di-ferric forms. EPR quantification of the $S={}^1/_2$ mixed valent form in the experiment above showed that it corresponded to ~30% of YtfE concentration. Fe^{3+} has much lower affinity for NO compared to the Fe^{2+} form, and so capacity to bind NO decreases as the di-iron site becomes oxidized. Furthermore, binding of NO to the mixed valent center (if this occurs) would result in a diamagnetic or integer spin, and thus EPR-silent, species.

An equivalent experiment to the above was carried out, except that air-exposed YtfE was used (~40% mixed valent form). Analysis revealed no detectable N_2O, ~10% mixed valent species and MNIC corresponding to <0.01 mol per mol of YtfE. The loss of mixed valent form of YtfE demonstrates that it can react with nitrite.

Thus, overall, much higher amounts of NO compared to N_2O are generated when di-ferrous YtfE reactions with nitrite, and the estimation of NO generated here is likely to be lower than the actual amount.

Bioinformatic analyses of YtfE and NO reductases. Protein BLAST searches of the NCBI RefSeq Select database for proteins similar to the *E. coli* K-12 YtfE, and the NO reductases Hcp, Hmp and NorV within the Enterobacteriaceae (taxid 543) were carried out. Significant alignments were taken as those with ≥35% sequence identity with ≥60% sequence coverage. This analysis yielded 90 different taxid hits for YtfE, 102 for Hcp, 110 for Hmp, and 102 for NorV. There were 11 taxid numbers that possessed YtfE but not Hcp, 15 that possessed YtfE but not Hmp, and 19 that possessed YtfE but not NorV. Of the 11 YtfE-plus taxids that lack Hcp, 7 possessed NorV. Of the remaining 4 taxids, 1 was Hmp-plus, leaving 3 taxids with an apparently orphaned YtfE (i.e. they apparently lack Hcp, Hmp and NorV). Thus, where Hcp is missing from YtfE-containing species, an alternative NO reductase is present in most cases. It is possible that an as yet uncharacterized NO reductase could be present in the few species that apparently lack any of the recognised NO reductases.

In summary, in the Enterobacteriaceae, NO production by YtfE is very often coupled to reduction to N_2O (or protein nitrosation) via Hcp. Where Hcp is missing, YtfE may function together with another type of NO reductase. Overall, it is reasonable to conclude that, in general, YtfE is functionally linked to Hcp or an alternative NO reductase.

SUPPORTING TABLES

Table S1. Predicted masses of YtfE species.

	**Species**	**Predicted Mass (Da)**	**Observed Mass (Da)**[a]	**ΔMass (Da)**
Wild type YtfE				
Apo	Apo $(RSH)_2$	26,030	26,030	0
	Apo (RS-SR)	26,028	-	-
Fe	$Fe^{(II)}$	26,084	26,085	+1
	$Fe^{(III)}$	26,083	-	-
Di-ferrous	$[Fe^{(II)}Fe^{(II)}O](RSH)_2$	26,156	26,154	-2[b]
	$[Fe^{(II)}Fe^{(II)}O]$ (RS-SR)	26,154	26,152 [c]	-2
Mixed valent	$[Fe^{(II)}Fe^{(III)}O]$ $(RSH)_2$	26,155	26,153	-2
	$[Fe^{(II)}Fe^{(III)}O]$ (RS-SR)	26,153	26,151	-2
Di-ferric	$[Fe^{(III)}Fe^{(III)}O]$ $(RSH)_2$	26,154	26,152 [a]	-2
	$[Fe^{(III)}Fe^{(III)}O]$ (RS-SR)	26,152	26,150	-2
MNIC	$[Fe^{(II)}Fe^{(II)}O]$ (NO) $(RSH)_2$	26,184	-	-
	$[Fe^{(II)}Fe^{(III)}O]$ (NO) $(RSH)_2$	26,185	26,183	-2
	$[Fe^{(II)}Fe^{(III)}O]$ (NO) (RS-SR)	26,183	-	-
DNIC	$(NO)_2$ $[Fe^{(II)}Fe^{(II)}O]$ $(RSH)_2$	26,216	-	-
	$(NO)_2$ $[Fe^{(II)}Fe^{(II)}O]$ (RS-SR)	26,214	-	-
$EthylNO_2$	$[Fe^{(II)}Fe^{(II)}O](C_2H_5ONO)(RSH)_2$	26,231	26,230	-1
Nitrite	$[Fe^{(II)}Fe^{(II)}O](NO_2^-)(RSH)_2$	26,202	-	-
	$[Fe^{(II)}Fe^{(III)}O](NO_2^-)(RSH)_2$	26,203	-	-
	$[Fe^{(II)}Fe^{(II)}O](NO_2^-)$(RS-SR)	26,200	26,199	-1
	$[Fe^{(III)}Fe^{(III)}O](NO_2^-)$(RS-SR)	26,198	-	-
C30A/C31A YtfE				
Apo		25,965	25,966	+1
Di-ferrous	$[Fe^{(II)}Fe^{(II)}O]$	26,091		
Mixed valent	$[Fe^{(II)}Fe^{(III)}O]$	26,090	26,088	-2

[a] Dash indicates not observed. [b] Charge compensation, involving dissociation of protons upon binding of a positively charged cofactor, is commonly observed for metalloproteins. The systematic shift of -2 Da in the observed mass compared to predicted mass for di-iron forms of YftE could indicate atypical charge compensation. [c] Note in some cases masses cannot distinguish between di-ferrous (RS-SR) and di-ferric $(RSH)_2$, or a mixture these species.

Table S2. Simulated EPR parameters.

Species	S	g-tensor	Linewidth (MHz)	Anisotropic residual linewidth (MHz)
MNIC	3/2	4.06, 3.95, 2.00	52	197, 209, 12
DNIC	1/2	2.02	67	-
Mixed valent YtfE	1/2	1.87, 1.91, 1.96	64	87, 100, 72

SUPPORTING FIGURES

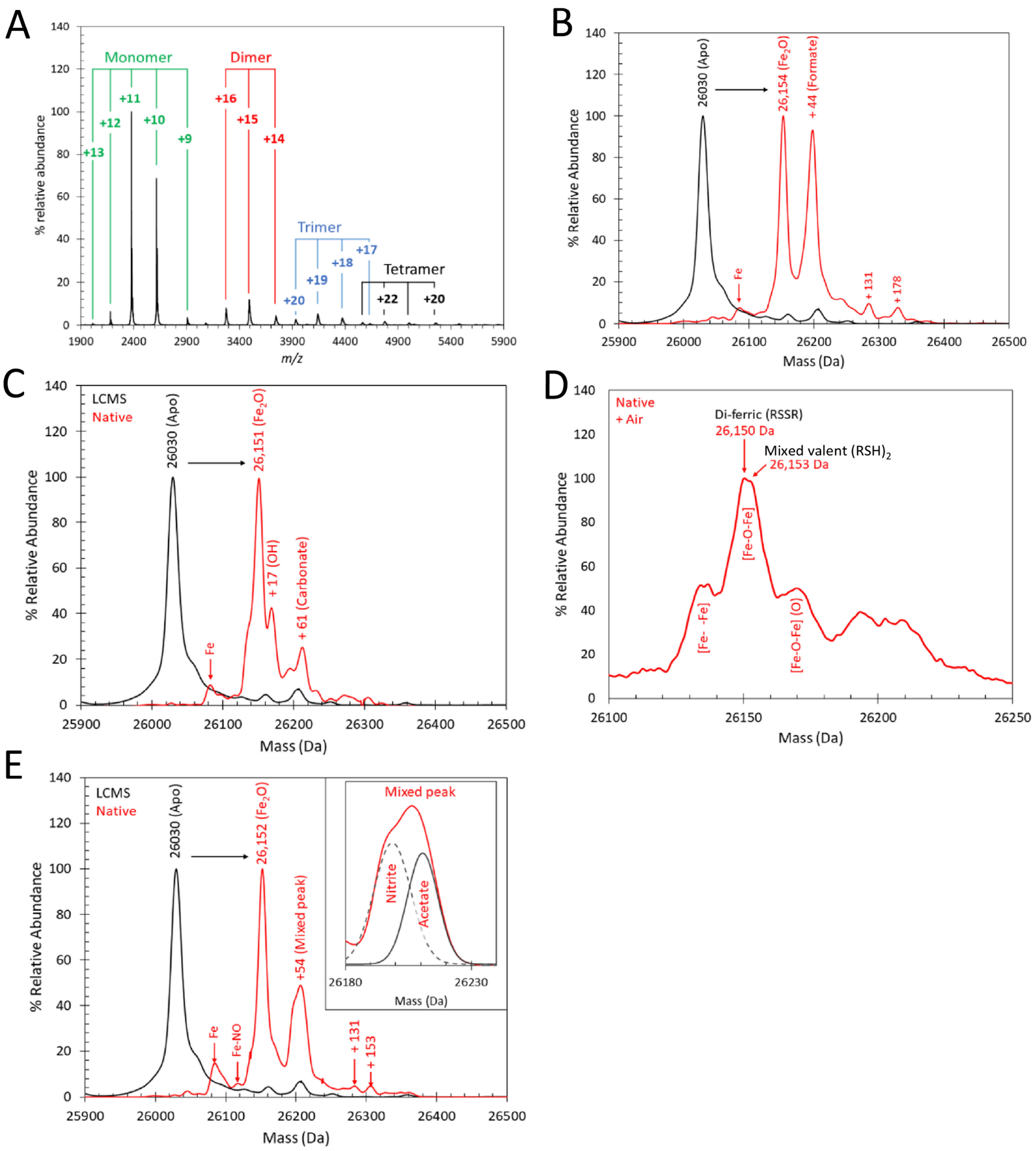

Figure S1. Additional MS and spectroscopy of YtfE. A) Native MS charge state distribution for as isolated, di-ferrous YtfE in positive mode is dominated by the monomeric form. Small amounts (~10%) of dimeric and higher order species are also observed. Deconvoluted native MS of YtfE ionized from B) ammonium formate or C) triethylammonium bicarbonate. D) Native MS confirms prolonged O_2 exposure results in di-ferric and mixed valent YtfE with varying levels of disulfide bond formation, as well as damage to the di-iron site (species with mass equivalent to loss of 1Fe atom). E) Native MS of YtfE isolated from $NaNO_2$-supplemented cells, revealing a mixture of YtfE redox states and some damage to the di-iron site as well as nitrite and NO adducts. Lower intensity peaks were also observed on both the high and low mass sides. Higher mass peaks at 26,285 and 26,308 Da corresponded to YtfE with incomplete N-terminal methionine residue removal (+131 Da) and its sodium adduct. Lower mass peaks at 26,084 and 26,114 Da corresponded to YtfE containing a single iron atom, and a possible Fe-NO species, respectively.

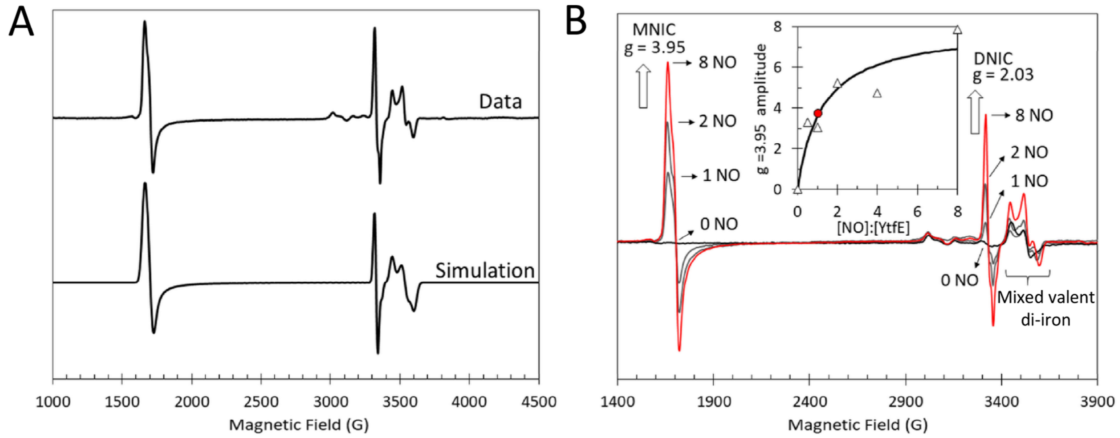

Figure S2. EPR studies of NO-treated YtfE. A) The EPR spectrum of YtfE following exposure to NO (Fig. 3D of main paper), featuring a S = ½ mixed valent di-iron site, S = $^3/_2$ MNIC and S = ½ DNIC signals, was fitted using Easyspin[16] (pepper) in Matlab. Given the intrinsic linewidth of ferric EPR spectra, hyperfine coupling frequencies were not simulated. Instead, anisotropic residual linewidth was used to account for differences in line broadening. Fitting outputs are given in Table S2. For the S = $^3/_2$ MNIC species, g-values indicate near axial symmetry, with E/D close to zero. B) Titration of di-ferrous YtfE with NO (as proliNONOate) resulted in increasing MNIC and DNIC signals; inset shows a plot of amplitude of MNIC signal as a function of the ratio of NO to YtfE. The MNIC signal observed after the addition of nitrite to di-ferrous YtfE (red point, see Fig. 4B) is shown for comparison.

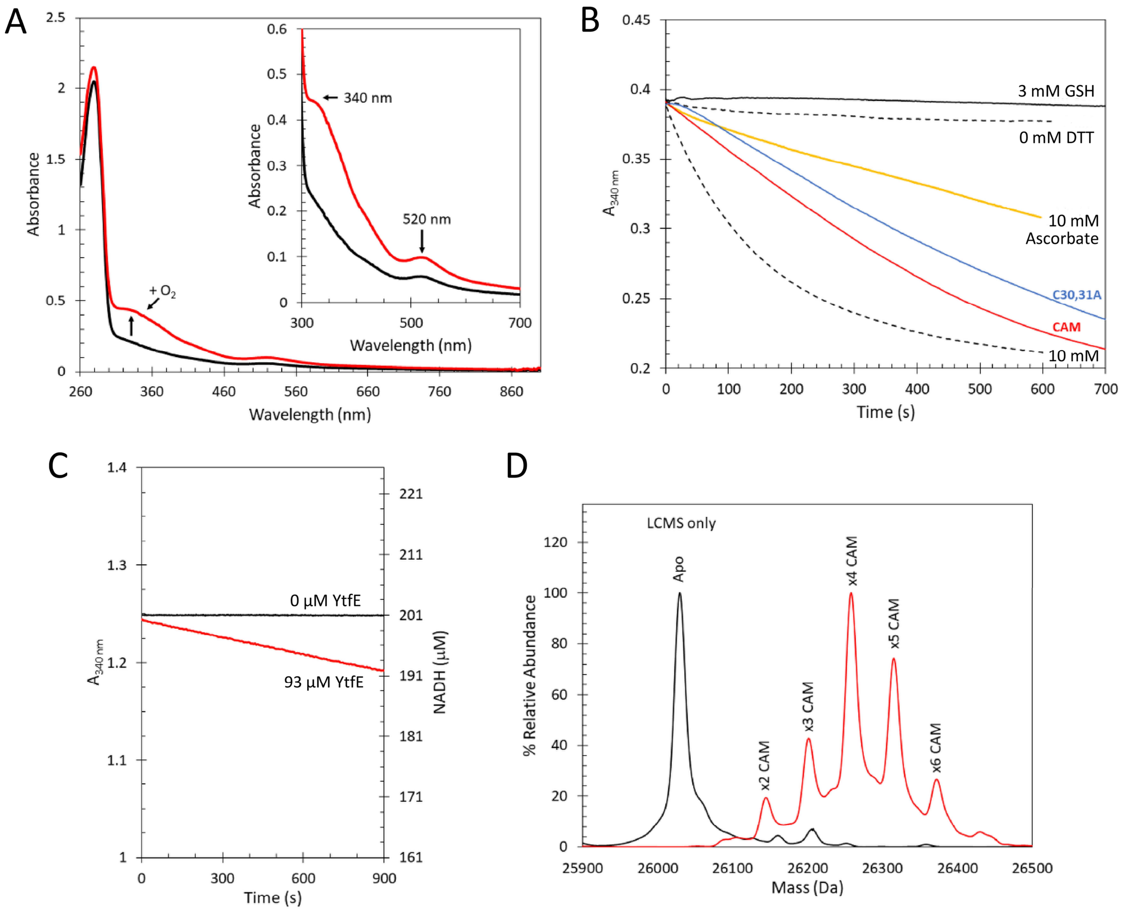

Figure S3. DTT reduction of mixed valent YtfE. A) DTT-reduced YtfE (black line) was exposed to air after desalting, resulting in the reappearance of absorbance bands at 340 and 520 nm; inset shows 300 - 700 nm region in more detail. B) Reduction of YtfE by glutathione (solid black line), ascorbate (yellow) and the effect of carboxymethylation (red line) or Cys30Ala/Cys31Ala substitutions (blue line) on DTT-mediated reduction. The response of YtfE in the presence and absence of 10 mM DTT is shown for comparison (dashed lines). C) The effect of YtfE on NADH oxidation. D) Denaturing LC-MS mass spectrum of YtfE before and after carboxymethylation. YtfE contains three cysteine residues (Cys30, Cys31 and Cys184) that could undergo reaction with iodoacetamide to become carboxymethylated (CAM). The spectrum shows an average of four (and minimum of two) CAM modifications per YtfE. We note that although iodoacetamide displays a high degree of specificity for thiols, it will, under certain conditions, result in additional modifications.[17]

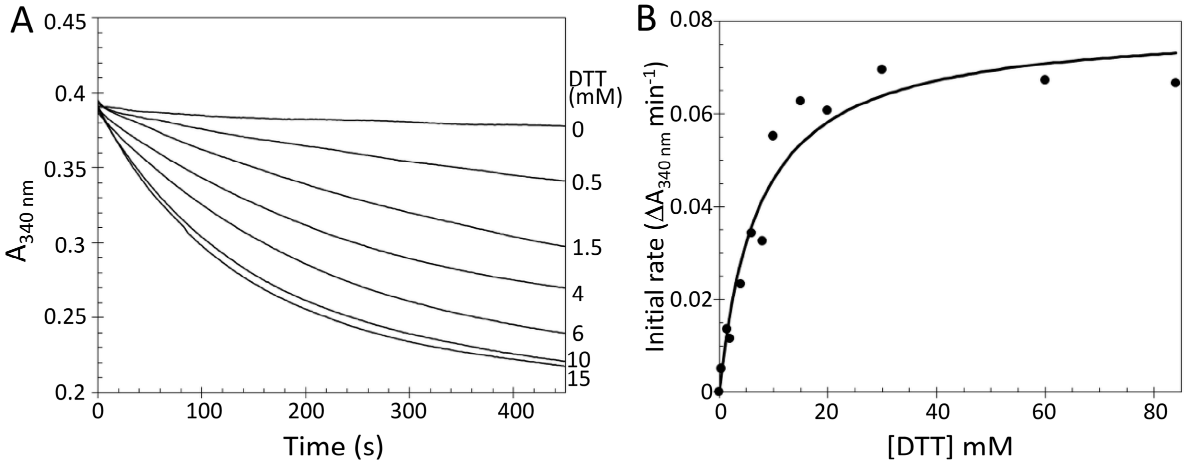

Figure S4. Reduction of YtfE by dithiothreitol (DTT). A) The kinetics of reduction of air exposed (mixed valent and di-ferric) YtfE (~90 μM) were measured by monitoring the decrease in absorbance at 340 nm after the addition of DTT to the indicated final concentrations. A clear dependence of the observed rate of reduction upon the concentration of DTT was observed. B) A plot of the initial rate ($\Delta A_{340\ nm}$ min^{-1}) as a function of DTT concentration. The solid line represents a binding isotherm, giving a K_d of 7.3 mM for DTT.

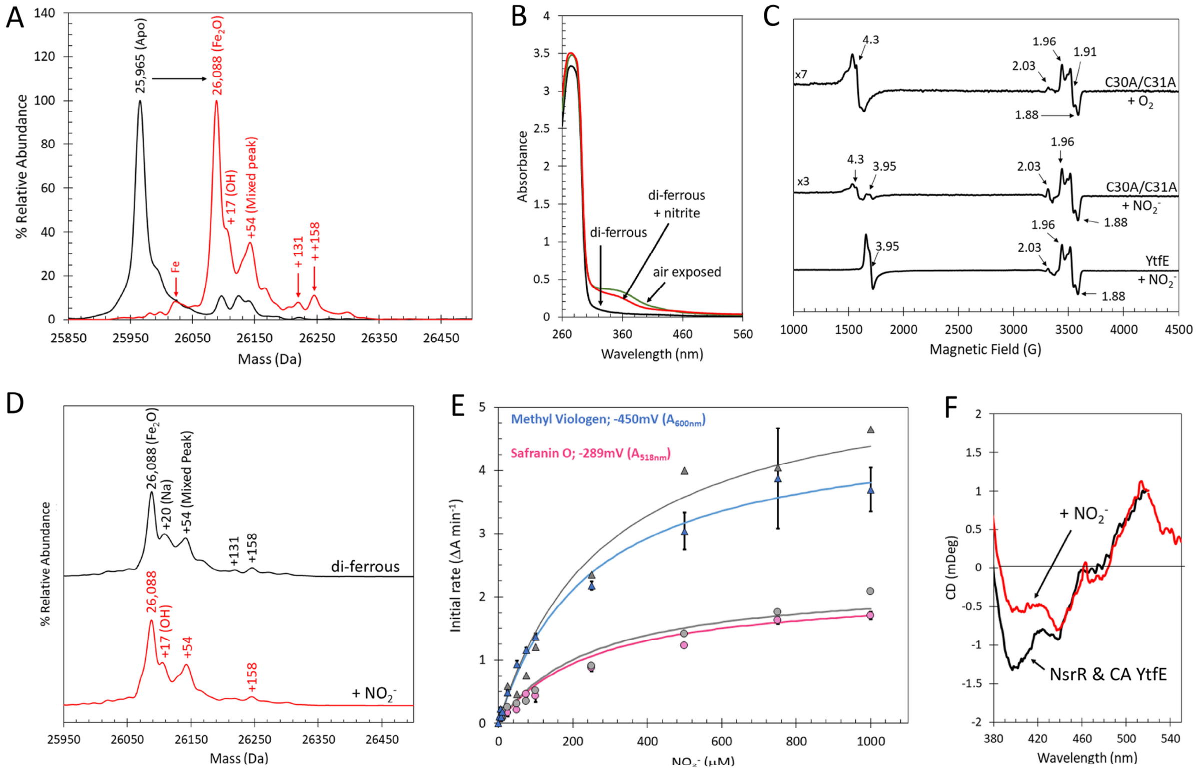

Figure S5. Further characterization of Cys30Ala/Cys31Ala YtfE. A) Native MS of the as isolated enzyme revealed hydroxide (+17 Da), nitrite and acetate adducts (mixed peak +54 Da) along with indication of oxidation of the di-iron site. B) Absorbance spectra of di-ferrous Cys30Ala/Cys31Ala YtfE (black line), and in the presence of nitrite (red line) or O_2 (green line) indicates that Cys30Ala/Cys31Ala YtfE cannot auto-nitrosylate. C) EPR spectroscopy of Cys30Ala/Cys31Ala YtfE in the presence of O_2, and nitrite confirms the inability of the double Cys variant to auto-nitrosylate. The response of wild type di-ferrous YtfE is shown for comparison. D) Deconvoluted native MS of Cys30Ala/Cys31Ala YtfE following the addition of nitrite. This provides further evidence of the lack of reaction with nitrite (compare Fig. 4C). E) Comparison of Cys30Ala/Cys31Ala YtfE-mediated oxidation of methyl viologen (gray triangles) and safranin O (gray circles) in comparison to wild type YtfE, blue triangles and pink circles, respectively. Error bars indicate standard deviation (SD) from the mean (where error bars are not visible, the SD was smaller than the data point symbol). YtfE proteins were 10 μM. For safranin O experiments, an increase in $A_{518\ nm}$ indicated safranin O oxidation, the rate of which was dependent upon the concentration of nitrite. Fits of initial rates (gray, blue, pink lines) using a simple Michaelis-Menten equation indicated comparable K_m values for nitrite, see Table 1 for kinetic parameters). F) CD spectra of di-ferrous Cys30Ala/Cys31Ala YtfE (46 μM) and [4Fe-4S] NsrR (20 μM) solution before (black line) and after the addition of 3 mM nitrite (red line). Only limited cluster damage was observed, consistent with a compromised auto-nitrosylation activity.

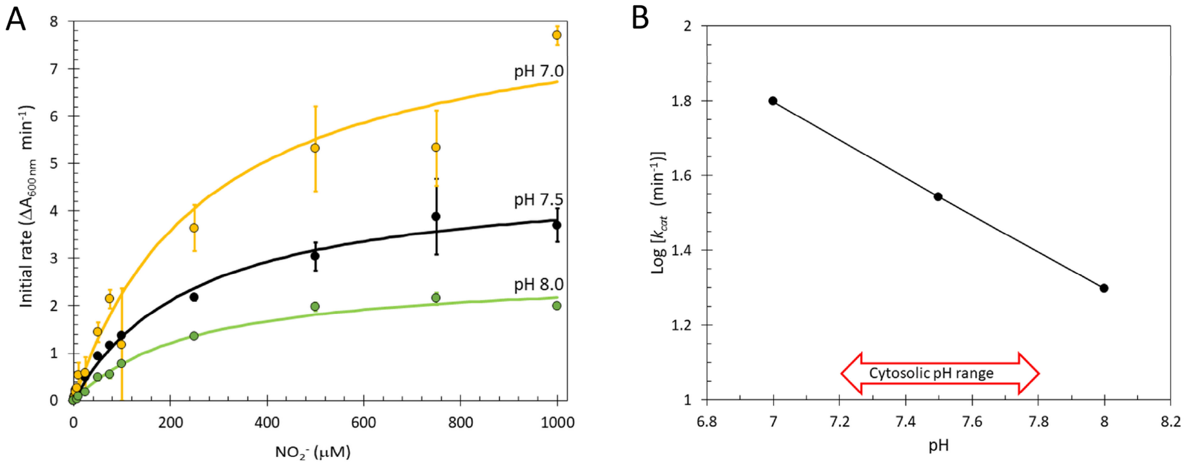

Figure S6. pH dependence of YtfE-catalysed nitrite reduction. A) Initial rates analysis; fits (yellow, black, green lines) to a simple Michaelis-Menten equation, see Table 1 for full kinetic parameters. Error bars indicate standard deviation (SD) from the mean (where error bars are not visible, the SD was smaller than the data point symbol). B) pH dependence of methyl viologen-mediated, YtfE-dependent, nitrite reduction over a physiological pH range. k_{cat} values were derived from the fits shown in A).

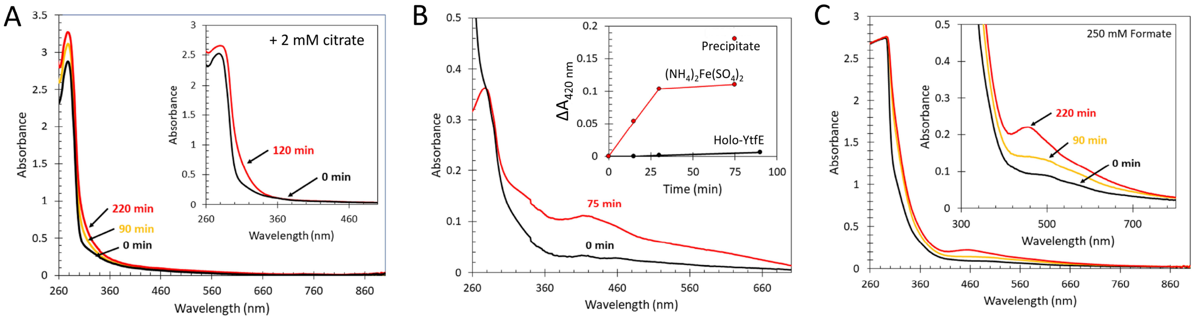

Figure S7. YtfE-mediated repair of apo-ferredoxin. A) Absorption spectra monitoring YtfE-mediated repair of apo-ferredoxin (25 μM) at 0 min (black line), 90 min (yellow line) and 220 min (red line), in the presence of 50 μM di-ferrous YtfE, 2.5 μM IscS, 10 mM DTT, 3 mM L-Cys. Inset: the same reaction in the presence of 2 mM citrate. B) Absorption spectrum of ferredoxin, before (black line) and after (75 min) reconstitution with Fe^{2+} salts. Inset: plot of $\Delta A_{420\ nm}$ using YtfE (black circles, line) or Fe^{2+} salts (red circles, line) as the source of iron. C) Absorption spectra of YtfE-mediated repair as in A), but in the presence 250 mM ammonium formate. Absorbance of ferredoxin, 0 min (black line), 90 min (yellow line) and 220 min (red line). Inset shows changes in more detail.

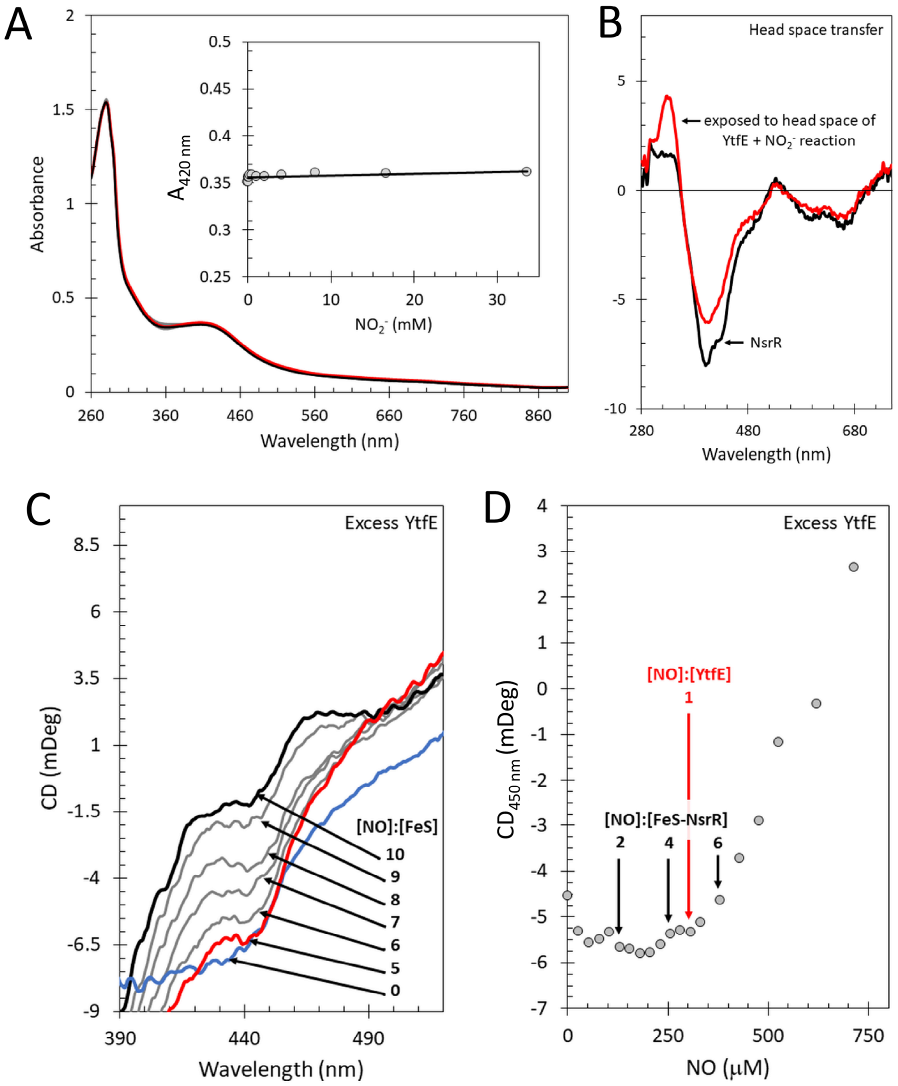

Figure S8. The effect of YtfE on [4Fe-4S] NsrR. A) [4Fe-4S] NsrR titrated with increasing amounts of nitrite; the inset shows changes in $A_{420\ nm}$ as a function of the nitrite concentration. B) CD spectrum of [4Fe-4S] NsrR before (black) and after (red) exposure to the head space of the YtfE-mediated nitrite reduction reaction. C) Difference CD spectra of a solution containing di-ferrous YtfE (300 μM) and [4Fe-4S] NsrR (65 μM, blue line). The (-)450 nm band indicative of [4Fe-4S] NsrR decreased between ~5 to 10 [NO]:[FeS], red and black lines, respectively. Intermediate NO ratios are in gray. D) Plot of CD intensity at 450 nm as a function of NO concentration. Damage to [4Fe-4S] NsrR occurred after ~1 NO per YtfE.

SUPPORTING REFERENCES

1. Balasiny, B.; Rolfe, M. D.; Vine, C.; Bradley, C.; Green, J.; Cole, J., Release of nitric oxide by the *Escherichia coli* YtfE (RIC) protein and its reduction by the hybrid cluster protein in an integrated pathway to minimize cytoplasmic nitrosative stress. *Microbiology* **2018,** *164* (4), 563-575.
2. Justino, M. C.; Almeida, C. C.; Goncalves, V. L.; Teixeira, M.; Saraiva, L. M., *Escherichia coli* YtfE is a di-iron protein with an important function in assembly of iron-sulphur clusters. *FEMS Microbiol Lett* **2006,** *257* (2), 278-284.
3. Garbett, K.; Darnall, D. W.; Klotz, I. M.; Williams, R. J., Spectroscopy and structure of hemerythrin. *Arch Biochem Biophys* **1969,** *135* (1), 419-434.
4. Munnoch, J. T.; Martinez, M. T.; Svistunenko, D. A.; Crack, J. C.; Le Brun, N. E.; Hutchings, M. I., Characterization of a putative NsrR homologue in *Streptomyces venezuelae* reveals a new member of the Rrf2 superfamily. *Sci Rep* **2016,** *6*, 31597.
5. Crack, J. C.; Volbeda, A.; Le Brun, N. E.; Fontecilla-Camps, J. C., Structure–Function Relationships of the NsrR and RsrR Transcription Regulators. In *Encyclopedia of Inorganic and Bioinorganic Chemistry*, 2020; pp 1-23.
6. Lo, F. C.; Hsieh, C. C.; Maestre-Reyna, M.; Chen, C. Y.; Ko, T. P.; Horng, Y. C.; Lai, Y. C.; Chiang, Y. W.; Chou, C. M.; Chiang, C. H.; Huang, W. N.; Lin, Y. H.; Bohle, D. S.; Liaw, W. F., Crystal structure analysis of the repair of iron centers protein YtfE and its interaction with NO. *Chemistry* **2016,** *22* (28), 9768-9776.
7. Johnson, K. A.; Verhagen, M. F.; Brereton, P. S.; Adams, M. W.; Amster, I. J., Probing the stoichiometry and oxidation states of metal centers in iron-sulfur proteins using electrospray FTICR mass spectrometry. *Anal Chem* **2000,** *72* (7), 1410-1418.
8. Stewart, M. Y. Y.; Bush, M. J.; Crack, J. C.; Buttner, M. J.; Le Brun, N. E., Interaction of the Streptomyces Wbl protein WhiD with the principal sigma factor sigma(HrdB) depends on the WhiD [4Fe-4S] cluster. *J Biol Chem* **2020,** *295* (28), 9752-9765.
9. Pellicer Martinez, M. T.; Crack, J. C.; Stewart, M. Y.; Bradley, J. M.; Svistunenko, D. A.; Johnston, A. W.; Cheesman, M. R.; Todd, J. D.; Le Brun, N. E., Mechanisms of iron- and O_2-sensing by the [4Fe-4S] cluster of the global iron regulator RirA. *Elife* **2019,** *8*, e47804.
10. Crack, J. C.; Le Brun, N., Mass spectrometric identification of [4Fe-4S](NO)x intermediates of nitric oxide sensing by regulatory iron-sulfur cluster proteins. *Chemistry* **2019**, *25*, 3675-3684.
11. Crack, J. C.; Thomson, A. J.; Le Brun, N. E., Mass spectrometric identification of intermediates in the O_2-driven [4Fe-4S] to [2Fe-2S] cluster conversion in FNR. *Proc Natl Acad Sci U S A* **2017,** *114* (16), E3215-E3223.
12. Lei, Q. P.; Cui, X.; Kurtz, D. M., Jr.; Amster, I. J.; Chernushevich, I. V.; Standing, K. G., Electrospray mass spectrometry studies of non-heme iron-containing proteins. *Anal Chem* **1998,** *70* (9), 1838-1846.
13. Frottin, F.; Martinez, A.; Peynot, P.; Mitra, S.; Holz, R. C.; Giglione, C.; Meinnel, T., The proteomics of N-terminal methionine cleavage. *Mol Cell Proteomics* **2006,** *5* (12), 2336-2349.
14. Geoghegan, K. F.; Dixon, H. B.; Rosner, P. J.; Hoth, L. R.; Lanzetti, A. J.; Borzilleri, K. A.; Marr, E. S.; Pezzullo, L. H.; Martin, L. B.; LeMotte, P. K.; McColl, A. S.; Kamath, A. V.; Stroh, J. G., Spontaneous alpha-N-6-phosphogluconoylation of a "His tag" in *Escherichia coli*: the cause of extra mass of 258 or 178 Da in fusion proteins. *Anal Biochem* **1999,** *267* (1), 169-184.
15. Averill, B. A.; Vincent, J. B., [2] Electronic absorption spectroscopy of nonheme iron proteins. In *Methods in Enzymology*, Academic Press: 1993; Vol. 226, pp 33-51.
16. Justino, M. C.; Almeida, C. C.; Teixeira, M.; Saraiva, L. M., *Escherichia coli* di-iron YtfE protein is necessary for the repair of stress-damaged iron-sulfur clusters. *J Biol Chem* **2007,** *282* (14), 10352-10359.
17. Stoll, S.; Schweiger, A., EasySpin, a comprehensive software package for spectral simulation and analysis in EPR. *J Magn Reson* **2006,** *178* (1), 42-55.
18. Yang, Z.; Attygalle, A. B., LC/MS characterization of undesired products formed during iodoacetamide derivatization of sulfhydryl groups of peptides. *J Mass Spectrom* **2007,** *42* (2), 233-243.
